# Effects of gestational perfluorohexanesulfonic acid exposure at human realistic dose on social communication deficit in mouse offspring

**DOI:** 10.1016/j.ebiom.2026.106160

**Published:** 2026-02-10

**Authors:** Shengmei Zhang, Chang Gao, Ruonan Li, Jia Lv, Jingjing Xu, Maohua Miao, Hong Liang, De-Xiang Xu, Bo Wang, Yichao Huang

**Affiliations:** a School of Public Health and Center for Big Data and Population Health of IHM, Anhui Medical University, Hefei, 230031, China; b Department of Pediatrics, Suzhou Hospital of Anhui Medical University, Anhui Medical University, Suzhou, 234099, China; c Shanghai-MOST Key Laboratory of Health and Disease Genomics, NHC Key Lab of Reproduction Regulation, Shanghai Institute for Biomedical and Pharmaceutical Technologies, Shanghai, 200237, China

**Keywords:** Perfluorohexanesulfonic acid, PFAS, Gestational chemical exposure, Social communication deficits, GABAergic signalling

## Abstract

**Background:**

Early-life exposure to exogenous chemicals can disrupt neurodevelopment. Perfluorohexanesulfonic acid (PFHxS), a legacy PFAS widely used and detected globally, remains poorly studied for its neurotoxicity.

**Methods:**

CD-1 mice (n = 90 dams) were exposed to human-relevant dose of PFHxS from gestational day (GD) 0–17. PFHxS levels were measured in maternal plasma and foetal/offspring medial prefrontal cortex (mPFC) (GD 18, postnatal weeks [PNW] 4 and 10) using liquid chromatography tandem mass-spectrometry (LC-MS/MS). Offspring social behaviour was assessed with the Three-Chamber social test. Neurotransmitters in mPFC of PNW 10 offspring were profiled by LC-MS/MS, transcriptomics was performed on GD 18 and PNW 4 mPFC, GABAergic neurons were quantified by immunofluorescence, and glutamate decarboxylase (GAD) expression by western blotting. PFHxS-GAD interactions were examined via molecular docking and microscale thermophoresis (MST).

**Findings:**

Maternal plasma reached 5.1 ± 0.1 ng/mL, equivalent to human biomonitoring data, and PFHxS accumulated in foetal mPFC (68.1 ± 4.1 pg/g). PFHxS exposure induced social deficits at PNW 4 and 10, which were more pronounced in males. The mPFC of PFHxS exposed offspring exhibited an excitatory-tilted neurotransmitter profile, feature with reduced γ-aminobutyric acid (GABA, 90.6 ± 12.2 vs. 70.2 ± 4.3 μg/g, *p* = 0.008). The differentially expressed genes were enriched in GABAergic and synaptic signalling pathways. Despite unchanged percentage of GABAergic neuron and GAD expression, metabolite GABA/glutamate ratio was attenuated, suggesting impaired GAD function. Both molecular docking and MST suggested moderate-to-strong binding affinity between PFHxS and GAD, affecting GABA synthesis.

**Interpretation:**

Gestational PFHxS exposure at human-relevant levels impairs offspring social behaviour, likely via disruption of GAD-mediated imbalance in excitation/inhibition.

**Funding:**

This work was provided by grants from the National Natural Science Foundation of China (82404221 and 82373586), Anhui Provincial Natural Science Foundation (2308085Y50 and 2408085QH275), Education Department of Anhui Province for Excellent Young Scientist (2022AH030076), and funding from Center for Big Data and Population Health of IHM (JKS2022020).

**Research in context**

**Evidence before this study**

Per- and polyfluoroalkyl substances (PFAS), as widespread environmental pollutants, may disrupt child neurodevelopment. However, the neurotoxicological effect of perfluorohexanesulfonic acid (PFHxS) is less studied. Literature revealed epidemiological studies suggesting potential associations between gestational PFHxS exposure and impaired social and communication skills in early childhood. However, to the best of our knowledge, no in vivo studies to date have investigated the neurotoxic potential of PFHxS under human-realistic doses.

**Added value of this study**

As a legacy PFAS listed under Annex A of the Stockholm Convention, PFHxS is widely detected in pregnant women worldwide, yet evidence linking it to neurodevelopmental toxicity remains limited. Using a human-realistic PFHxS exposure dose, we demonstrate that gestational PFHxS exposure induces social deficits in offspring, with more pronounced effects in males. We further show that PFHxS penetrates and accumulates in the foetal brain, disrupting glutamate decarboxylase activity and inducing an excitation/inhibition imbalance in the medial prefrontal cortex. These findings suggest previously unrecognized neurotoxicity of PFHxS, which may persist into adulthood.

**Implications of all the available evidence**

Early life exposure to PFHxS may predispose the pups to altered GABAergic signalling during brain development. Our experimental data, together with existing human epidemiological evidence, suggest that PFHxS may impair long-term neurodevelopment in children, particularly in social communication. These findings advance our understanding of how chemical pollutant exposures may contribute to disease susceptibility and reinforce the importance of regulatory oversight to safeguard population health.

## Introduction

Per- and polyfluoroalkyl substances (PFAS), a class of persistent organic pollutants widely distributed in the environment, are known for their heat, water, and oil resistance and thus extensively used in industrial production and consumer products.^1^ In recent years, increasing awareness of PFAS accumulation in the environment and biological systems, as well as their potential toxicological effects, has drawn global public health concern.^2^ Perfluorohexanesulfonic acid (PFHxS), a representative PFAS, has been widely applied in industrial and consumer products, resulting in human and environmental exposure.^3^ More importantly, PFHxS has been estimated to have a biological half-life between 4.7 and 15.5 years,^4^ and can cross major biological barriers within human body, including the placental barrier^5^ and blood–brain barrier.^6^ In 2022, it was added to Annex A of the Stockholm Convention owing to concerns regarding its persistence, bioaccumulation, and potential toxicity.^7^ Evidence from both epidemiological investigations and experimental studies substantiates the systemic toxicity of PFHxS, indicating significant impacts on overall health, including hepatic,^8^^,^^9^ reproductive,^10^^,^^11^ and immune functions.^12^ Prior studies have consistently demonstrated that PFHxS is detected in nearly all pregnant women across various regions and ethnic groups, with median concentrations reported to be around 1 ng/mL.^13^, ^14^, ^15^, ^16^ Mounting epidemiological evidence links gestational PFHxS exposure to adverse outcomes across multiple health domains (e.g., birth outcomes, cardiovascular health, overweight and obesity),^13^^,^^17^^,^^18^ yet its potential developmental neurotoxicity in children is still not clearly established.

Social deficiency is a hallmark shared across diverse neuropsychiatric disorders.^19^ Autism spectrum disorder (ASD) is characterised by difficulties in social communication and interaction and by restricted repetitive behaviours. The evidence regarding the association between prenatal PFHxS exposure and ASD is inconclusive. While certain studies have reported an increased risk^20^ and others have suggested a potential protective effect,^21^ findings from larger cohort investigations have not demonstrated consistent associations,^22^ which may be attributable to variations in study design, sample size, and exposure assessment methods. Several longitudinal studies from China assessed neurobehavioral development repeatedly during infancy and early childhood,^23^, ^24^, ^25^, ^26^ and three of which found that prenatal PFHxS exposure was linked to an increased risk of low communication and personal-social developmental trajectories.^23^, ^24^, ^25^ Another prospective study also showed that prenatal exposure to PFHxS was associated with increased risk of social deficits at age four, an association that appeared stronger in genetically susceptible children.^27^

To date, very few studies have specifically investigated the neurotoxicity of PFHxS, particularly under realistic scenarios in mammals. Previous research using zebrafish embryo found that PFHxS induced hyperactivity of the adult fish at nonteratogenic concentrations.^28^ In a mice model, neonatal exposure to PFHxS at higher than environmentally relevant-dose led to long-term memory impairment in adult mice via down-regulation of growth associated protein-43 and calcium/calmodulin dependent protein kinase II.^29^ The neurotoxic effects of gestational PFHxS exposure on social communication deficits remain unexplored in vivo, and its underlying mechanisms are largely unknown. Here, we investigate how human-relevant gestational PFHxS exposure affects social and behavioural outcomes in mouse offspring, and uncover potential mechanistic pathways.

## Methods

### Animal experiments

Eight-week-old specific-pathogen-free (SPF) CD-1 adult mice (both male and female) were purchased from Beijing Vital River Laboratory Animal Technology Co., Ltd., (Beijing, China). The mice underwent seven days of acclimation and were subsequently housed at a 2:1 female-to-male ratio for mating. Gestational day (GD) 0 was confirmed by the presence of vaginal plugs. Pregnant mice (n = 90) were randomly divided into three groups: control (corn oil [Aladdin, China, 8123971] containing 0.03% dimethyl sulfoxide [DMSO, MedChemExpress, USA; HY-Y0320, CAS No. 67-68-5; ≥99:0]), 0.03 (PFHxS-Low), and 0.3 (PFHxS-High) μg/kg/d PFHxS (Macklin, China; P850157, CAS No. 355-46-4; ≥95%) in 0.03% DMSO. The human-realistic dose was derived from our previous publication,^30^ plasma PFHxS concentration of pregnant mice exposed to PFHxS-High dose fall in the range observed in human biomonitoring programme.^31^ All test substances were administered via oral gavage once a day from GD 0 to GD 17. Half of the dams were sacrificed at GD 18 using intraperitoneal sodium pentobarbital (100 mg/kg) to collect foetal medial prefrontal cortex (mPFC). Maternal blood was collected and plasma was separated after centrifugation at 3500 rpm for 15 min. The remaining pregnant mice underwent spontaneous vaginal delivery for assessing autism-like behaviour in the offspring at postnatal week (PNW) 4 and PNW10, respectively. A protocol specifying the primary endpoints and core analyses was prepared prior to study initiation but was not preregistered in a publicly accessible registry. For blinding, group allocation and treatment administration were performed by different personnel, although the latter was unblinded due to procedural requirements. The outcome assessors were blinded to treatment, and an infrared tracking system was used to minimise potential bias. Data analysis was conducted under partial blinding. There were no a priori inclusion or exclusion criteria for animal handling or data analysis.

### Behavioural assessment

Given the developmental milestones of mice, with PNW 4 representing early adolescence and PNW 10 corresponding to young adulthood,^32^ neurobehavioral assessments were conducted at these stages (n = 10 per sex per group per time point) to evaluate both the early-life and enduring effects of in utero adversities. The mPFC of offspring mice at PNW 4 and 10 were collected after completion of behavioural assessment.

The Three-Chamber social interaction apparatus was used to assess sociability and social novelty in mice. This apparatus consists of a large rectangular box divided into three interconnected chambers: a central chamber flanked by two side chambers. Each containing an opening that can be closed off by a door to control access. For sociability test, the testing mouse was first placed in the middle chamber, while an unfamiliar mouse (Stranger 1, S1) matched for age and sex was placed in one other chamber on the side and leaving the other chamber empty (E). Social behavioural trajectory and sniffing time in S1 or E was recorded by behaviour software (Smart 3.0, RWD, China). Social index was determined through the following equation: (S1 - E)/(S1 + E). For social novelty test, the testing mouse was first gently guided back to the middle chamber, after which a second unfamiliar mouse (Stranger 2, S2) was introduced into the previously empty chamber. Upon opening the side doors, the sniffing times directed towards the familiar mouse (S1) or the novel mouse (S2) were simultaneously recorded. Social preference index was determined through (S2 – S1)/(S1 + S2). Sniffing time (sec) was used as an index of social inclination, with normally developing mice expected to spend more time sniffing S1 during the sociability phase and more time sniffing S2 during the social novelty phase.

Throughout the experiments, temperature, humidity, and light-noise conditions were kept consistent. After testing each animal, the cages were cleaned with 75% ethanol to reduce scent interference in subsequent experiments.

### Determination of PFHxS concentration in maternal plasma and offspring's mPFC

Concentration of PFHxS in maternal plasma (GD 18) and the offspring's mPFC was quantified at GD 18, PNW 4 and 10 (n = 5 per group per timepoint) using liquid chromatography-tandem mass spectrometry (LC-MS/MS, AB SCIEX 5500, USA). The mPFC of the mice was first homogenised in 300 μL ultrapure water. PFHxS quantification was performed using either 50 μL of plasma or 200 μL of mPFC homogenate. PFOA (20 μL, 10 ng/mL; P815842, Macklin Biochemical, China; CAS No. 335-67-1; ≥96%) was added as internal standard for quantification purpose. After thorough vortex, 1 mL methanol (1060351000, Merck, USA; CAS No. 67-56-1; ≥99.9%) was added for PFHxS extraction. The mixture was vortexed and sonicated for 20 min prior to centrifugation at 10,000 rpm for 7 min to collect supernatant. Another 500 μL methanol was added to the original vial for repeat extraction. Supernatant from two-rounds of extraction was combined and further purified using solid-phase extraction (Oasis HLB, 1 cc, 30 mg sorbent; Waters, USA). The eluted solvent was further dried under nitrogen and then reconstituted in 150 μL methanol prior to injection. PFHxS in mPFC was analysed with LC-MS/MS system equipped with a Zorbax Eclipse Plus C18 column (50 mm × 2.1 mm, 1.8 μm particle size). Instrumental information is available in Table S1. To minimise background contamination, all vials and inserts used in the experiment were rinsed three times with ethanol (≥95%) before use. For quality assurance and quality control, recovery tests were performed using blank spikes and matrix spikes. The recovery rates for PFHxS were 90.2 ± 0.05% and 84.4 ± 0.08% in blank and brain tissue, respectively (Table S1). A procedural blank was included and processed together with the samples to assess any potential laboratory or environmental contamination. Only trace amount of PFHxS and PFOA were detected in the blank, indicating minimal background interference (Figures S1 & S2). Final PFHxS concentration in the experimental samples were adjusted by subtracting the background level.

### Determination of neurotransmitters and related metabolites in the mPFC

Neurotransmitters are central to neuronal communication and the maintenance of normal brain function, and imbalances, particularly between excitatory and inhibitory signalling, have been implicated in various neurological disorders. The concentration of targeted metabolites in the mPFC were determined by LC-MS/MS (n = 5 per group per timepoint for both sexes). First, approximately 50 mg of mPFC tissue was homogenised in 300 μL cold saline. For extraction of metabolites, 800 μL of extraction solvents (acetonitrile/methanol, v/v = 1/1; 1000291000, Merck, USA; CAS No. 75-05-8; ≥99.9%) containing L-Phenyl-d5-alanine-2,3,3-d3 internal standard (5 μg/mL; HY-N0215S12, Toronto Research Chemicals, Canada) was added to 200 μL homogenate, the mixture was vortexed for 5 min. After incubation at −20 °C for 2 h and centrifugation for 10 min (14,000 rpm, 4 °C), the supernatant was collected and then dried under gentle nitrogen flow, which was later reconstituted in 150 μL solvent (acetonitrile/water, v/v = 1/1) for LC-MS/MS analysis. Chromatographic separation was performed on an ACQUITY Premier HSS T3 column (1.8 μm, 2.1 × 150 mm; Waters ACQUITY UPLC, USA).^30^ Instrumental information is available in Table S3.

### RNA sequencing and data analysis

RNA sequencing was conducted to characterise gene expression and elucidate molecular pathways underlying the observed phenotypes. To reduce batch effects, mPFC tissues from GD 18 and PNW 4 male offspring (n = 3 per group per time point) were processed together. The mPFC tissue were first homogenised in TRIzol® Reagent (15596026, Thermo Fisher Scientific, USA) for total RNA extraction. RNA integrity was verified by Agilent 5300 Bioanalyzer (Agilent Technologies, USA; RIN >9.0), and purity/concentration was measured via NanoDrop 2000 spectrophotometer (Thermo Fisher Scientific, USA; A260/A280 = 1.8–2.2). Subsequently, cDNA libraries were prepared with TruSeq™ RNA Library Prep Kit (FC-122-1001, Illumina, USA). Libraries were quantified using the Qubit® 4 Fluorometer and validated with the QuantiFluor® dsDNA System (E2671, Promega, USA). The sequence was performed on the NovaSeq 6000 platform using NovaSeq 6000 S2 Reagents (20028314, Illumina, USA) to generate 150-bp paired-end reads. Raw reads were filtered by fastp (https://github.com/OpenGene/fastp) (v0.23.2, default parameters) to remove low-quality sequences (Q < 20) and adaptor contamination, yielding high-quality clean reads.^33^ The clean reads were aligned to the mouse reference genome GRCm39 using HISAT2^34^ (v2.1.0) (http://ccb.jhu.edu/software/hisat2/index.shtml) with >90% mapping rate. Differentially expressed genes (DEGs) were identified by DESeq2 (v1.40.2) (http://bioconductor.org/packages/stats/bioc/DESeq2/) with thresholds of |fold change| >1.2 and *p* < 0.05. Reactome and Gene Ontology (GO) analyses were performed using ReactomePA version 1.44.0 (http://bioconductor.org/packages/release/bioc/html/ReactomePA.html) and KOBAS version 3.0.0 (http://bioinfo.org/kobas/download/), and software, respectively. Gene set variation analysis (GSVA) was conducted to compute sample-level pathway enrichment scores, and differential pathway activity between groups was subsequently assessed using the limma linear modelling framework.^35^

### Western blot for glutamate decarboxylase (GAD) 67/65

To investigate the regulation of GABA synthesis, the expression of key rate-limiting enzymes in GABA synthesis, the GAD isoforms, was quantified using western blotting. The mPFC tissues of male mice at GD 18 (n = 3 per group per time point) were homogenised in RIPA lysis buffer (P0013C, Beyotime Biotechnology, China) and then centrifuged at 12,000 rpm for 15 min. The supernatant was taken for protein quantification according to the bicinchoninic acid method. Approximately 6 μg of protein were separated by sodium dodecyl sulphate-polyacrylamide gel electrophoresis (SDS-PAGE, PG112, Yeasen Biotechnology, China) and subsequently transferred to a polyvinylidene fluoride (PVDF) membrane. After blocking in 5% milk for one and half hours, the membrane was first incubated with primary antibody (rabbit GAD65/67, AB183999, Abcam, Cambridge, UK, RRID: AB_3662875) overnight at 4 °C, and then with corresponding secondary antibody (1:10,000) for 2 h at room temperature. Protein bands were visualised using chemiluminescence. Signal intensity of target bands was analysed using Image J, with *β* - actin (200068-8F10, ZenBio, China) as the loading control for normalisation. Original uncropped western blotting with all bands is available in a supplementary file.

### Immunofluorescence

Immunofluorescence was used to quantify GAD-expressing GABAergic neurons in the mPFC, providing a direct assessment of potential pathological changes. Following brain tissue perfusion, the brains of PNW 4 and 10 male offspring mice (n = 3 per group) were dehydrated with sucrose and embedded in optimal cutting temperature compound. Brain sections were cut to a thickness of 30 μm. In each group, three to four mPFC sections at the same position were permeabilised with 0.5% Triton X-100 in phosphate-buffered saline (PBS) for 1 h at room temperature. Sections were first blocked with 10% normal goat serum 1 h, before overnight incubation with primary antibodies (rabbit GAD65/67, AB183999, RRID: AB_3662875, Abcam, Cambridge, UK) at 4 °C. Following three rounds of PBS wash, the sections were then incubated with species-matched fluorescent secondary antibodies for 2 h under light-protected conditions at room temperature. Finally, tissue sections were treated with DAPI-containing anti-fade mounting medium. The numbers of GAD 65/67 neurons were analysed by panoramic tissue cell quantitative analysis system (TissueFAXS Plus S, Austria).

The anti-GAD65/67 antibody (AB183999, Abcam, RRID: AB_3662875) was validated for western blotting and immunofluorescence based on manufacturer data and prior study demonstrating GABAergic neuron-specific staining in experimental mouse model.^36^

### Molecular docking analysis

Molecular docking simulations were conducted using Molecular Operating Environment (MOE, 2022; Chemical Computing Group, Canada) to investigate the potential binding modes and affinities of PFHxS with GAD isoforms (GAD65: DCE2_HUMAN; GAD67: DCE1_HUMAN). Binding sites were identified via the MOE Site Finder module. Docking was performed using the induced-fit Triangle Matcher protocol with the AMBER10:EHT force field. Initial poses were scored with the London δ G function (top 10,000 retained), refined via rigid receptor fitting, and the top 100 poses were rescored using GBVI/WSA δ G. The optimal geometry was selected based on ligand interaction patterns. Conformation clustering classified dominant binding modes into 8 groups (GAD65) and 7 groups (GAD67), with the highest-affinity conformation selected for interaction mapping. All structural files (optimised receptors, ligand poses, interaction diagrams) were archived to ensure reproducibility. Binding affinities < −7 kcal/mol are considered strong, < −5 kcal/mol moderate, and > −5 kcal/mol indicate no predicted binding.^37^

### Microscale thermophoresis

To further validate the molecular docking results, microscale thermophoresis (MST) was employed to examine the binding affinity and stability between PFHxS and GAD65. MST detects molecular interactions by measuring changes in the motion of fluorescently labelled molecules within a microscopic temperature gradient, which is altered upon ligand binding.^38^ Mouse GAD65 (HY–P75178, MedChemExpress, USA) was purified and labelled with protein labelling kit RED-NHS 2nd Generation following the manufacture's instruction (NanoTemper Technologies, Germany). The labelled GAD65 and PFHxS (Shanghai Macklin Biochemical Co., Ltd, China) was loaded into MO-KO22 capillary column and scanned with Monolith NT.115 (NanoTemper Technologies, Germany).

### Ethics

All experimental procedures were carried out in accordance with the regulations of the Anhui Medical University Animal Care and Use Committee and Use of Laboratory Animals (LLSC20211179, Hefei, China). Animal Research: Reporting of In vivo Experiments (ARRIVE) were also followed.

### Statistics

The sample size was determined in accordance with the relevant guidelines of the Organisation for Economic Co-operation and Development (OECD) and the International Council for Harmonisation (ICH), which recommend using 10 litters per dose group, allowing for the selection of one male and one female pup per litter for the neurotoxicity assessments.^39^^,^^40^ Considering the major study endpoints, relative brain weight at GD 18, PNW 4, and PNW 10, as well as the behavioural testing at PNW 4 and PNW 10, we targeted for 15 litters per dose group per endpoint. This number was selected to ensure that at least 10 litters would be available for evaluation after accounting for possibility of false pregnancy due to reliance on visual inspection of vaginal plug, and potential losses during gestation and the postnatal period. Thus, a total of 90 pregnant animals were included to ensure adequate sample retention throughout the experimental period.

All experimental data are presented as Mean ± Standard Error of the Mean (S.E.M.) from at least three independent biological replicates. The Shapiro–Wilk test was applied to assess data normality. Two-tailed Student's t-tests were used for pairwise comparisons. For multiple-group comparisons, one-way ANOVA was applied followed by Tukey's post hoc test to evaluate between-group differences. Above mentioned statistical analyses were performed with SPSS 23.0 (IBM, USA), respectively. For neurotransmitters, the concentrations were first log_10_-transformed and mean-centred, and then scaled by the standard deviation of each metabolite prior to further analysis. Partial Least Squares Discriminant Analysis (PLS-DA), heatmap, and Variable Importance in Projection (VIP) plot were generated with MetaboAnalyst 6.0 (https://www.metaboanalyst.ca/). Statistical significance was set at two-tailed *p* < 0.05. False discovery rate was controlled using the Benjamini–Hochberg method, and *q* < 0.10 was regarded as statistically significant.

### Role of funders

The funding bodies were not involved in the design of the study, the collection of data, the analysis of data, the interpretation of results, or the writing of the manuscript.

## Results

### Gestationally exposed PFHxS accumulates in foetal mPFC

**Fig. 1:**
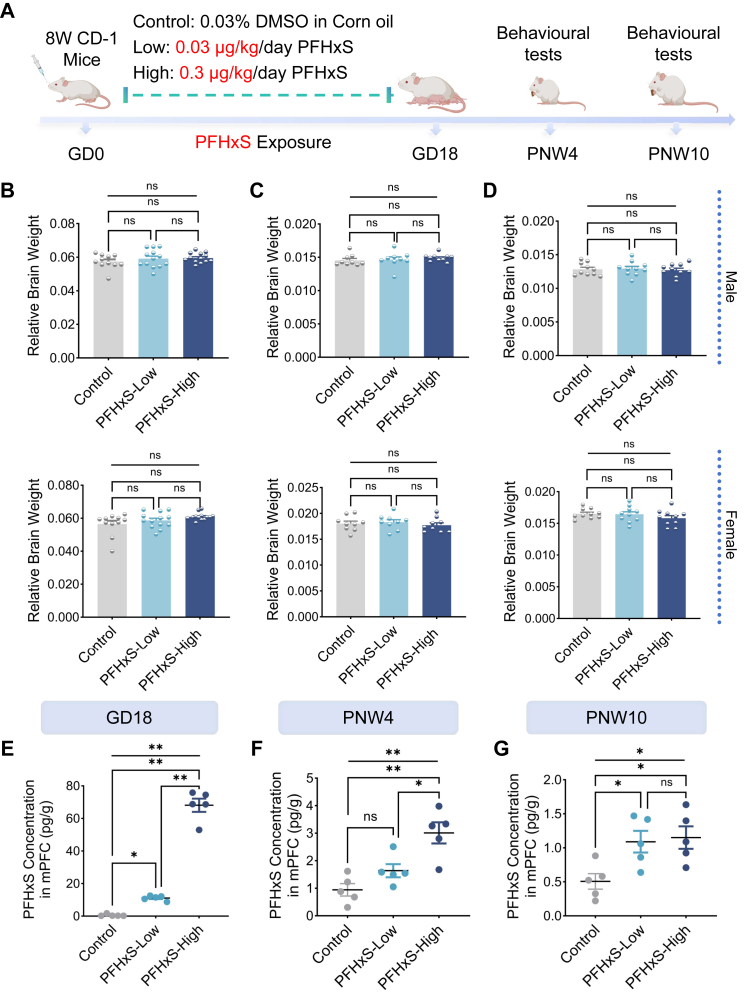
**Gestational PFHxS exposure accumulates in the foetal and offspring medial prefrontal cortex (mPFC) without affecting relative brain size**. (A) Pregnant CD-1 mice were administered with corn oil containing 0.3% DMSO (control), or PFHxS dissolved in corn oil containing 0.3% DMSO at 0.03 μg/kg/d (PFHxS-Low) and 0.3 μg/kg/d (PFHxS-High), from GD 0–17 (n = 30/group). (B–D) Relative brain weight was measured at GD 18 foetus and PNW 4/10 offspring (n = 9–13/sex/group). (E–G) Concentration of PFHxS was measured in the mPFC of control and PFHxS-exposed mice (n = 5/group/time point). Statistical analysis was performed with ANOVA with Tukey's post hoc analysis, asterisk indicating the statistically significance level, where ∗*p* < 0.05, ∗∗*p* < 0.01. Data are displayed as mean ± S.E.M.

Pregnant mice were exposed to a human-relevant dose of PFHxS during GD 0–17 to observe its impact on the neurobehaviors of offspring (Fig. 1A). Statistically significant differences in plasma PFHxS concentration were found at GD 17 (*p*_Overall_ < 0.0001 [ANOVA], Table S4). Plasma PFHxS concentration was minimal in control dam (0.08 ± 0.02 ng/mL), while that reached 2.03 ± 0.40 and 5.14 ± 0.13 ng/mL in dams of PFHxS-Low and PFHxS-High group, respectively (Table S4). No differences in relative brain weight were observed between the control group and either treatment group at any time point in either sex (Fig. 1B–D, Table S5). However, a dose-dependent accumulation of PFHxS in the mPFC was observed in foetal mice and offspring of exposed dams at GD18 and PNW4, where statistically significant differences were observed between mice of PFHxS-Low and PFHxS-High group (Fig. 1E–G, Table S6). The difference in concentration of PFHxS in the mPFC between PFHxS-Low and PFHxS-High group at PNW 4 (1.6 ± 0.2 vs. 3.0 ± 0.4 pg/g, *p* = 0.015 [ANOVA with Tukey's test]) was smaller than that observed at GD18, and no statistical differences were observed between the two groups at PNW 10 (1.1 ± 0.2 vs. 1.2 ± 0.2 pg/g, *p* = 0.956 [ANOVA with Tukey's test]). For offspring of PFHxS-High group, concentration of PFHxS in foetal mPFC was at 68.1 ± 4.1 pg/g, which gradually decreased to 3.0 ± 0.4 and 1.2 ± 0.2 pg/g at PNW 4 and 10, respectively (Table S6).

### Gestational PFHxS exposure causes male-predominant social deficits

**Fig. 2:**
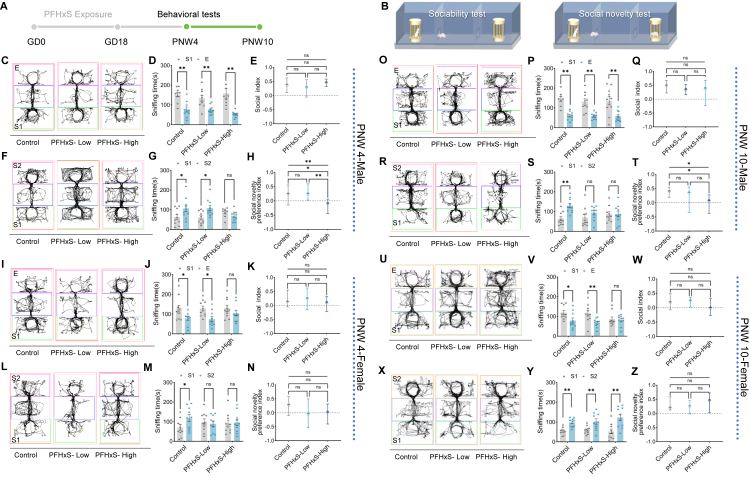
**Gestational PFHxS exposure causes consistent and pronounced social deficits in male offspring as assessed by the Three-Chamber Social Test.** (A) Pregnant CD-1 mice were fed on corn oil containing 0.3% DMSO (control), or PFHxS dissolved in corn oil containing 0.3% DMSO at 0.03 μg/kg/d (PFHxS-Low) and 0.3 μg/kg/d (PFHxS-High). (B) The Three-Chamber social test was performed on PNW 4 and 10 offspring to assess sociability and social novelty (n = 10/sex/group/time point). Social behavioural trajectory and sniffing time towards stranger mice and empty chamber was recorded with Smart 3.0 software. Social index was derived from [(stranger 1 - empty cage)/(stranger 1 + empty cage)], and social novelty preference index was calculated as [(stranger 2 - stranger 1)/(stranger 1 + stranger 2)]. (C, F, I, L, O, R, U, X) Behaviour trajectory tracked by the software. (D, G, J, M, P, S, V, Y) Differences in sniffing time between stranger 1 and E in sociability test, and between stranger 1 and 2 in social novelty test. (E, H, K, N, Q, T, W, Z) Differences in social index and social novelty preference index between control and PFHxS-exposed groups. Statistical analysis was performed with Student's t-test or ANOVA with Tukey's post hoc analysis, asterisk indicating the statistically significance level, where ∗*p* < 0.05, ∗∗*p* < 0.01. Data are displayed as mean ± S.E.M.

Three-Chamber social tests were conducted at PNW 4 and 10 to assess alterations in the social behaviour of offspring mice (Fig. 2A and B). At PNW 4, male offspring born to control and exposed dam of two dose groups showed a preference for S1 in sociability test (Fig. 2C and D, Table S7, *p* < 0.05 [Student's t-test]). No differences in the social preference index across three groups were observed (Fig. 2E–Table S8, *p*_Overall_ = 0.848 [ANOVA]). However, male offspring of PFHxS-High group showed no preference for S2 mouse in social novelty test (Fig. 2F and G, Table S7, sniffing time towards S1: 101.1 ± 15.6 s, S2: 69.6 ± 9.2 s, *p* = 0.101 [Student's t-test]). Compared to male offspring born to control dam, those of PFHxS-High group showed a significant reduction in social novelty preference index (Fig. 2H–Table S8, *p*_Overall_ = 0.003 [ANOVA], *p*_C__ontrol_ _vs. High_ = 0.009 [Tukey's test], *p*_Low_ _vs. High_ = 0.006 [Tukey's test]). Female offspring in the PFHxS-High group showed no inclination toward S1 during the sociability test (Fig. 2I and J, Table S9, sniffing time towards S1: 130 ± 15.5 s; E: 105 ± 13.7 s, *p* = 0.233 [Student's t-test]), although the social preference index did not differ significantly (Fig. 2K–Table S10, *p*_Overall_ = 0.516 [ANOVA]). In the social novelty phase, females from both PFHxS groups exhibited reduced interest in S2, yet the novelty preference index remained unchanged (Fig. 2L–N, Table S10, *p*_Overall_ = 0.149 [ANOVA]).

Similar patterns were observed at PNW 10 in male offspring. In the sociability test, mice displayed a strong social preference for S1, and the social index did not differ significantly between control and exposed groups (Fig. 2O–Q, Tables S11–S12, *p*_Overall_ = 0.571 [ANOVA]). In contrast, male offspring of both PFHxS exposed group showed declined social inclination towards S2 mouse (sniffing time towards S1: 87.1 ± 12.3 s; S2: 88.1 ± 14.8 s, *p* = 0.962 [Student's t-test]), and the social novelty preference index was significantly reduced in those of PFHxS-High group (Fig. 2R–T, *p*_Overall_ = 0.027 [ANOVA], *p*_C__ontrol_ _vs. High_ = 0.020 [Tukey's test]). Nonetheless, in female offspring from the PFHxS-High group, we observed no significant difference in sniffing time between S1 and E, but a significant difference between S1 and S2, indicating impaired sociability but preserved social novelty (Fig. 2U–Z, Tables S13 and S14, sniffing time towards S1: 86.4 ± 10.9 s; E: 81.3 ± 8.69 s, *p* = 0.717 [Student's t-test]; S1: 52.6 ± 12.0 s, S2: 123 ± 16.4 s, *p* = 0.003 [Student's t-test]).

Overall, these findings suggest that exposure to a human-realistic dose of PFHxS (0.3 μg/kg/d) induces significant social behaviour deficits, primarily through impaired social novelty preference, with males showing greater and more consistent susceptibility.

### PFHxS-induced social deficits is tied to excitatory/inhibitory (E/I) imbalance

**Fig. 3:**
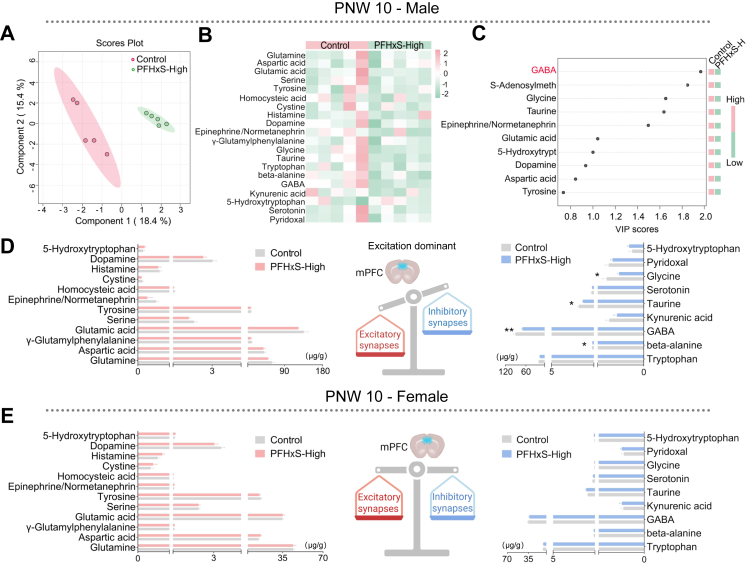
**PFHxS exposure-induced social deficits are tied to imbalanced excitatory-to-inhibitory neurotransmitter profile**. (A) Differences in the medial prefrontal cortex (mPFC) neurotransmitter profiles between male offspring from control and PFHxS-High group using Partial Least Squares Discriminant Analysis (PLS-DA) (n = 5/group). (B) Heatmap showing differences in the concentration of neurotransmitters and related metabolites between male offspring from control and PFHxS-High group (n = 5/group). (C) The top 10 metabolites were displayed with Variable Importance in Projection (VIP) score plot (n = 5/group). (D–E) Differences in the concentration of excitatory and inhibitory neurotransmitters in mPFC of control and PFHxS-High exposed male and female offspring, respectively (n = 5/sex/group). Statistical analysis was performed with Student's t-test, multiple comparisons were controlled with Benjamini-Hochberg method. Asterisk indicating the statistically significance level, where ∗*q* < 0.10. Data are displayed as mean ± S.E.M.

The neurotransmitter profile in male offspring differed significantly between control and PFHxS-High mice (Fig. 3A and B), with GABA contributing most to the separation (Fig. 3C). Analysis of absolute concentrations revealed no significant changes in excitatory neurotransmitters; however, several inhibitory neurotransmitters, including GABA (control: 90.6 ± 12.2 μg/g; PFHxS-High: 70.2 ± 4.3 μg/g, *p* = 0.008 [Student's t-test], *q* = 0.072), taurine (control: 1.9 ± 0.3 μg/g; PFHxS-High: 1.4 ± 0.1 μg/g, *p* = 0.017 [Student's t-test], *q* = 0.074), and glycine (control: 0.2 ± 0.04 μg/g; PFHxS-High: 0.1 ± 0.01 μg/g, *p* = 0.033 [Student's t-test], *q* = 0.074), were significantly reduced in the PFHxS-High group, resulting in an imbalanced excitatory/inhibitory network (Fig. 3D–Table S15). In contrast, in PNW 10 female offspring without impaired social function, no statistically significant differences in the neurotransmitter profiles were observed between control and PFHxS-High exposed groups (Fig. 3E–Table S16).

### Gestational PFHxS exposure disturbs the GABAergic signalling pathway

**Fig. 4:**
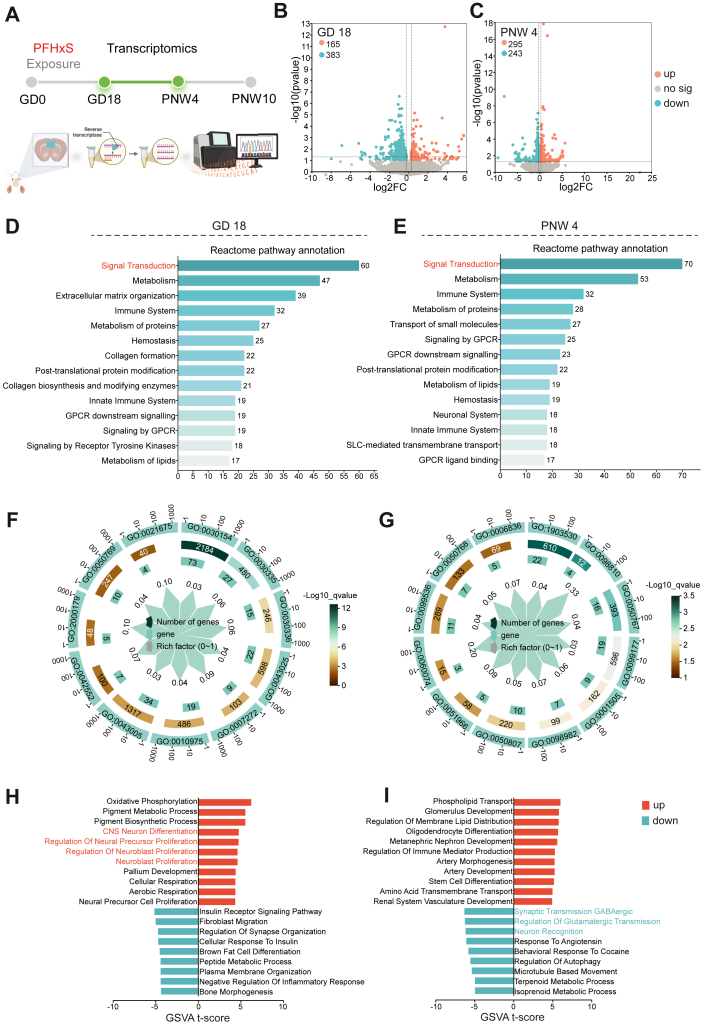
**Gestational PFHxS exposure disturbs the GABAergic signalling pathway in early life**. RNA sequencing was performed on the medial prefrontal cortex (mPFC) of GD 18 foetus and PNW 4 offspring from the control and PFHxS-High groups (n = 3/group/time point). (A) Experimental flowchart; (B–C) Volcano plot showing the differentially expressed genes (DEGs) between the two groups (|Fold change| >1.2, *p* < 0.05). (D–E) Reactome pathway annotation. (F–G) Gene Ontology (GO) enrichment of DEGs. (H–I) Gene set enrichment analysis (GSVA).

We then sought to explore gestational PFHxS exposure induced changes at transcriptomic level in mPFC of male offspring at GD 18 and PNW 4 of PFHxS-High group (Fig. 4A). At GD 18, compared to control mice, there were 383 down-regulated genes and 165 up-regulated genes in PFHxS exposed mice (Fig. 4B–Table S17). Similarly, 538 DEGs between control and exposed mice were found at PNW 4, with 243 down-regulated while 295 up-regulated, respectively (Fig. 4C–Table S18). Among these DEGs, 60 and 70 DEGs were mapped to signalling transduction pathway via Reactome at GD 18 and PNW 4, respectively (Fig. 4D and E, Tables S19–S20).

To identify the alterations in signalling pathways associated with mPFC biological processes and cellular components following PFHxS exposure, the pathway enrichment analysis of DEGs was performed using GO database. At GD18, PFHxS-exposed mice showed dysregulation of genes involved in the regulation of neuron projection development (GO:0010975), neuronal projection (GO:0043005), and positive regulation of neurogenesis (GO:0050769) compared with control mice (Fig. 4F–Table S21). In contrast, at PNW4 (Fig. 4G–Table S22), DEGs between treated and control mice were enriched in pathways related to the regulation of trans-synaptic signalling (GO:0099177), GABAergic synapses (GO:0098982), and neurotransmitter transport (GO:0006836). GSVA revealed that, relative to controls, the treated group exhibited enrichment of up-regulated pathways involved in neuronal differentiation and proliferation at GD18, whereas pathways related to GABAergic and glutamatergic synaptic signalling were down-regulated at PNW4 (Fig. 4H and I, Tables S23–S24).

### Gestational PFHxS exposure does not alter percentage of GABAergic cells and GAD expression

**Fig. 5:**
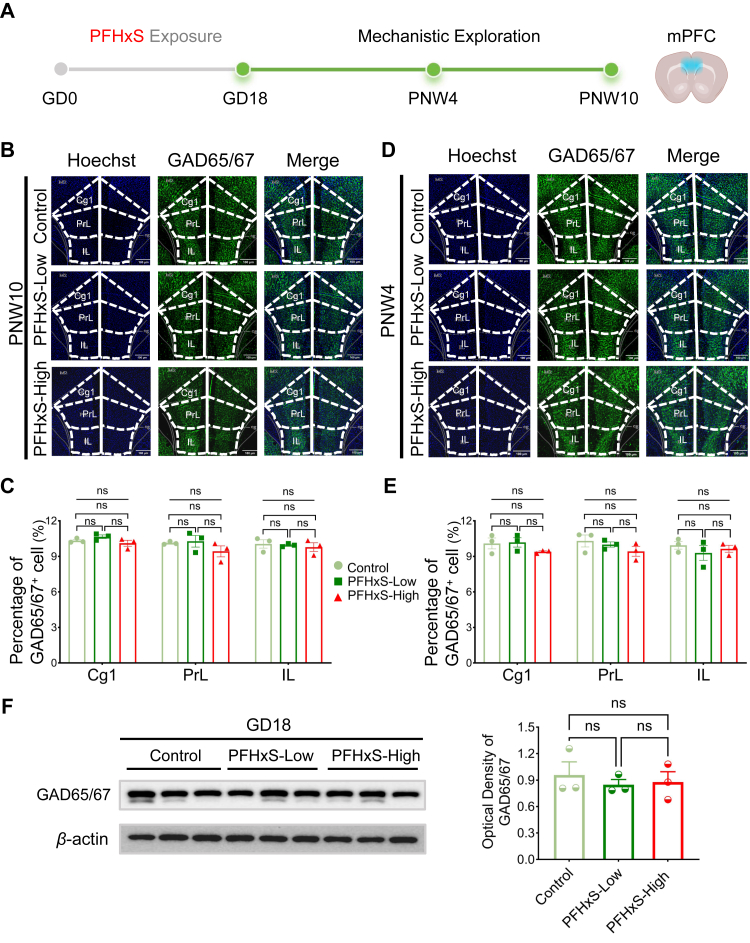
**Gestational PFHxS exposure induces social deficits without altering the percentage of GABAergic cells or glutamate decarboxylase (GAD) expression**. (A) Exploration of mechanisms were performed in the medial prefrontal cortex (mPFC) of GD 18 foetus and PNW 4/10 offspring (n = 3/group/time point). (B and D) Immunofluorescence imaging of GABAergic neurons in the mPFC of male offspring at PNW 4 and 10, showing distinct regions: cingulate cortex (Cg1), prelimbic cortex (PrL), and infralimbic cortex (IL) (n = 3/group). The original magnification was 400 × . (C and E) Percentage of GABAergic cells in mPFC of male offspring at PNW 4 and 10 in different regions (n = 3/group). (F) Expression of GAD in mPFC of GD 18 foetus by western blotting (n = 3/group). Statistical analysis was performed using ANOVA with Tukey's post hoc analysis, asterisk indicating the statistically significance level, where ∗*p* < 0.05, ∗∗*p* < 0.01. Data are displayed as mean ± S.E.M.

Since PFHxS-induced changes centred on GABAergic signalling, we next examined its impact on changes in GABAergic neurons of mPFC (Fig. 5A). In mPFC of PNW 10 male offspring, no differences in the percentage of GAD65/GAD67^+^ cells were found between control and exposed groups in any of the measured mPFC regions (Fig. 5B and C, Table S25). Specifically, in cingulate cortex (Cg1, control: 10.33%; PFHxS-Low: 10.65%; PFHxS-High: 10.11%, *p*_Overall_ = 0.178 [ANOVA with Tukey's test]), prelimbic cortex (PrL, control: 10.12%; PFHxS-Low: 10.28%; PFHxS-High: 9.42%, *p*_Overall_ = 0.332 [ANOVA with Tukey's test]), and infralimbic cortex (IL, control: 10.05%; PFHxS-Low: 9.95%; PFHxS-High: 9.78%, *p*_Overall_ = 0.819 [ANOVA with Tukey's test]). Similar findings were observed in male offspring at PNW 4 (Fig. 5D–E, Table S25, Cg1: *p*_Overall_ = 0.298 [ANOVA with Tukey's test]; PrL: *p*_Overall_ = 0.346 [ANOVA with Tukey's test]; IL: *p*_Overall_ = 0.602 [ANOVA with Tukey's test]). In GD 18 foetal mPFC, the relative expression of GAD isoforms was 0.95 ± 0.20, 0.81 ± 0.09, and 0.88 ± 0.12 of control, PFHxS-Low and PFHxS-High group, and no statistically significant differences were found overall or in-between groups (Fig. 5F–Table S26, *p*_Overall_ = 0.791 [ANOVA with Tukey's test]).

### PFHxS directly binds to and inhibits the activity of GAD

**Fig. 6:**
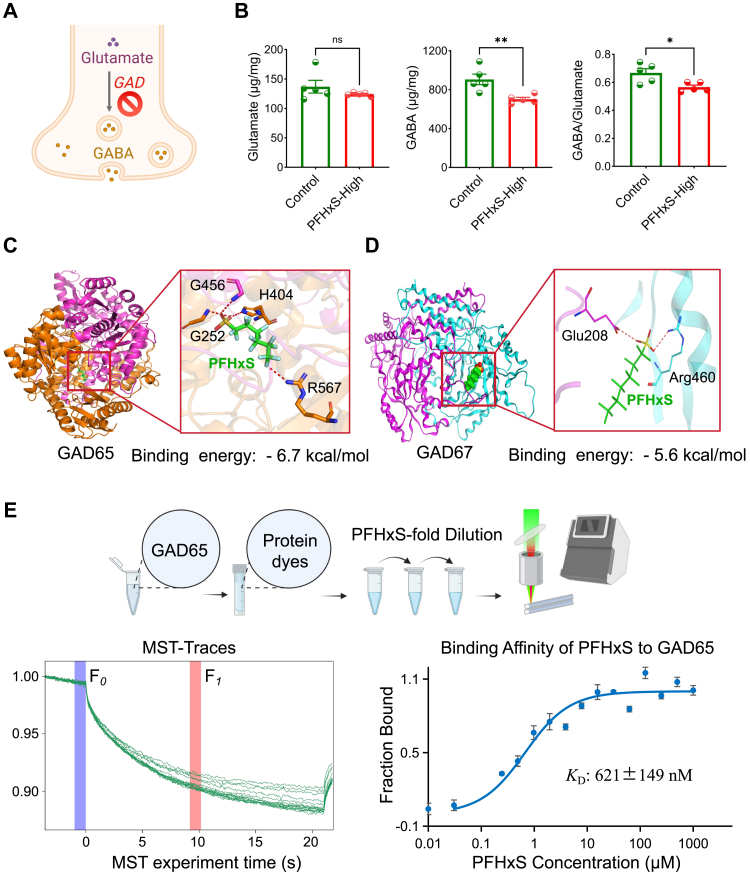
**Gestational PFHxS exposure induces social deficits in offspring through inhibiting glutamate decarboxylase (GAD) activity**. (A) A simplified pathway diagram of neurotransmitter conversion, depicting the process where glutamic acid is converted into GABA under the action of GAD. (B) Differences in the GABA, glutamate and GABA-to-Glutamate ratio between control and PFHxS-exposed offspring at PNW 10 (n = 5). (C–D) Computational prediction binding energies between GAD65/GAD67 proteins and PFHxS using molecular docking. (E) Measured binding affinity between PFHxS and GAD65 by microscale thermophoresis (MST). *F*_0_ denotes baseline fluorescence signal and *F*_1_ represents the fluorescence signal at thermophoretic state. Binding curve between PFHxS and GAD65 to generate disassociation constant (*K*_D_) (n = 3 independent experiments).

At PNW 10, GABA was significantly reduced in male PFHxS-High offspring, while glutamate remained unchanged, resulting in a lower GABA/glutamate ratio (0.69 ± 0.03 vs. 0.57 ± 0.02, *p* = 0.017 [Student's t-test], Fig. 6A and B, Table S27), implying a direct impairment of GAD. Based on molecular docking technique, a moderate-to-strong binding affinities were predicted for PFHxS and both GAD isoforms, where the binding energy was estimated to be −5.6 kcal/mol and −6.7 kcal/mol for GAD 67 and GAD 65, respectively (Fig. 6C and D). Moderate binding affinity (*K*_D_ 621 ± 149 nM) between PFHxS and GAD65 was also validated through MST (Fig. 6E).

## Discussion

Despite the widespread use and detection of PFHxS in environmental and biological matrices, the consequences of gestational exposure on offspring neurodevelopment remain poorly understood. In our animal model, continuous exposure to PFHxS during gestation (0.3 μg/kg/d, GD 0–17) resulted in human-realistic plasma levels (5.1 ± 0.1 ng/mL), which leads to accumulation in the foetal mPFC (68.1 ± 4.1 pg/g). PFHxS-exposed offspring exhibited significant social deficits in the Three–Chamber test at PNW 4 and 10, with effects more consistent and pronounced in males. We first demonstrated an imbalanced E/I ratio in the mPFC of PFHxS-High exposed group, featured predominantly by the reduction in the primary inhibitory neurotransmitter, GABA. Further, transcriptomic analysis indicated a disrupted GABAergic signalling, in line with the observation that PFHxS induced social deficits is centred around the GABAergic pathway. We further showed that although the number GABAergic neurons and the expression of two GAD isoforms were unchanged, a reduced GABA-to-glutamate ratio suggested impaired GAD function in PFHxS-exposed group. Molecular docking demonstrated moderate-to-strong binding affinities between PFHxS and both GAD isoforms, which was also confirmed by MST. Collectively, these findings show that gestational PFHxS exposure induces persistent social deficits by impairing GAD activity in the mPFC, leading to E/I imbalance. This study provides in vivo evidence that gestational PFHxS exposure disrupts sociability associated with impairing GAD activity in the mPFC, with effects persisting into adulthood.

Although PFHxS has recently been listed under the Stockholm Convention, it has not yet been completely phased out from production and continuous monitoring of its long-term health consequences are warranted. The global production of PFHxS increased nearly ten folds between 1985 and 2015,^41^ making it among the top five most detected PFAS in the environment and human biomonitoring programmes. Historically, in early 2000s, the mean concentration of PFHxS ranged between 2.0 and 7.6 ng/mL in the Australian HBM, while 1.1–3.2 ng/mL measured as part of the National Health and Nutrition Examination Survey (NHANES).^42^ A study gathered HBM data on PFHxS of 9 countries and regions^43^ found a global average of 0.61 ng/mL, with the highest concentration found in pregnant women of Poland (median: 2.29, IQR: 1.66–3.40 ng/mL), followed by Ukraine (median: 1.56, IQR: 0.98–2.24 ng/mL), and Sweden (median: 1.23, IQR: 0.80–1.99 ng/mL). Administration of PFHxS at 0.3 μg/kg/d yielded an animal model with a plasma concentration of 5.1 ng/mL, reflecting human-relevant exposure levels observed in the German population.^31^ Gestationally exposed PFHxS accumulated in foetal brain over the course of pregnancy, which drastically declined postnatally. Although it could partially be attributed to the growth of brain due to volume dilution, it must be noted that the biological half-life of PFHxS is much shorter in experimental animals.^44^ Taken together, the administered dose falls within the range of human-relevant exposure, and when considered alongside the substantially longer biological half-life in humans relative to experimental models, it may entail persistent health implications.

Using an in vivo model, we showed that gestational PFHxS exposure impairs social behaviour in offspring, with stronger and more consistent effects in males. Male offspring exposed to high PFHxS exhibited reduced sniffing time toward novel strangers and a consistently lower social novelty preference index at both PNW 4 and 10, indicating clear social deficits. Alternatively, female offspring showed reduced sniffing time toward strangers, yet their social index and social novelty preference remained unaffected, highlighting a male-predominant vulnerability to PFHxS exposure. In population studies, sex-specific differences in the social deficits have been observed across different neurodevelopmental disorders. In patients diagnosed with ASD, female are with more sophisticated social skills than males, but is still lower than that of typically developing children.^45^ Other studies also suggest greater social motivation and more frequent social interactions in autistic girls than in autistic boys.^46^^,^^47^ An in vivo study investigating sex-specific effects on social behaviour revealed consistent male–female differences across three inbred and outbred strains, most notably in the social novelty preference test.^48^ PFAS are known to induce sex-specific results across a range of health outcomes, largely due to their endocrine-disrupting properties. Following gestational exposure to PFOA and other endocrine-disrupting chemicals, either individually or in low-dose combination, neurobehavioral toxicity has been reported exclusively in male offspring, with no detectable effects in females.^49^ However, the mechanisms underlying these sex-dependent differences remain unclear. In one study, male mice acutely exposed to a single dose of PFHxS (6.1 or 9.2 mg/kg) on postnatal day 10 exhibited a significant elevation of cortical tau levels four months later, a pattern not observed in similarly exposed females.^50^ In contrast, gestational exposure to PFHxS (17 or 50 mg/kg/d) significantly reduced serum triiodothyronine and thyroxine levels in offspring rats; although no alterations in brain thyroid hormone signalling or sex-specific effects were detected.^51^ Similarly, epidemiological evidence suggests that prenatal PFAS co-exposure associated suboptimal development in communication is more pronounced in boys,^24^ though another study found girls are more affected.^23^

A review published in 2024 summarised the neurotoxicity of PFAS to be targeting on both the structure and function of brain, as well as secondary to effect on other organs, including the gut–brain axis.^52^ Dysregulation of the E/I balance has been widely recognised as a core pathological mechanism in neuropsychiatric disorders.^53^^,^^54^ In an autism-like animal model associated with *TRIO* GEF1 hypofunction, restoration of the E/I balance ameliorated social deficits via reactivation of GABAergic signalling.^55^ Our study demonstrated that PFHxS-induced social deficits in male offspring were mediated by disruption of GABAergic signalling, characterised by reduced GABA levels and a consequent shift toward an enhanced E/I balance in the mPFC. GABA plays a particularly crucial role due to its primary function as inhibitory signals,^56^ but GABA only represents a small fraction of the neurotransmitter network. Both legacy PFAS have been shown to affect E/I balance through altering the function of GABA-A receptors in human neuronal cells, consequently impairing behavioural and cognitive functions.^57^ Moreover, increasing body of evidence suggest that PFAS exposure not only affects the GABAergic system but may also induce E/I imbalance by modulating other neurotransmitters, including acetylcholine.^52^ Beyond direct modulation of these chemical messengers, PFAS has been shown to affect intracellular Ca^2+^ level by regulating the expression of Ca^2+^/calmodulin-dependent protein kinase II^58^ and the release and uptake of Ca^2+^ from the extracellular environment.^59^ Disrupted intracellular Ca^2+^ homoeostasis might then subsequently affect neurotransmitter release at synapses. In addition, female offspring in PNW 10 with minor social deficits showed no E/I imbalance, indicating that there may be other mechanisms involved in the social deficits induced by PFHxS.

Our work further demonstrated that PFHxS increases the E/I balance in mPFC and result in social deficits in the offspring via direct binding to the GABA synthesis rate-limiting enzyme, GAD. Although GABAergic neuron numbers and two GAD isoforms expression were unchanged, our findings show that PFHxS binds to GAD isoforms and results from MST further validated a moderate binding affinity between PFHxS and GAD 65.^60^ Despite the direct binding to GAD isoforms, gestational PFHxS exposure might exert similar effect in affecting GAD activity through other potential pathways. We have previously reported that gestational exposure to a human-relevant dose of PFHxS induces abnormal RNA alternative splicing, primarily through exon skipping. This splicing alteration affects RNA integrity and, consequently, the activity of the encoded proteins, even if their expression levels remain unchanged.^30^ Early work has also shown that post-translational modification (PTM) of GAD 67 through cAMP-protein kinase A (PKA) dependent pathway has been implicated in its enzyme activity and sequential GABA production.^61^ Both PFOA and PFOS reduced intracellular Ca^2+^ concentrations, which in turn lowered cAMP levels and consequently suppressed PKA activity in vitro.^62^ Although evidence is lacking for PFHxS, recent research has shown that PFOS could induces phosphorylation in Alzheimer's Disease related proteins.^63^ Other PFAS induced PTM events remain largely unexplored, especially in the nervous system.

This study features several unique strengths. Employing a human-realistic exposure enhances the clinical significance of the finding, offering a new insight into the environmental pollution related origin of health and diseases. Moreover, the social behaviour assessment was performed at two timepoints postnatally, offering insights to the long-lasting adverse effect of PFHxS into adulthood. However, limitations of this study should also be acknowledged. First, the investigation into the mechanisms underlying PFHxS-induced social deficits was limited to male offspring, due to the subtle and inconsistent phenotypes observed in females. While this likely reflects a sex-specific effect of PFHxS, further studies may be needed to clarify the underlying mechanisms in female offspring. Second, in the molecular docking analysis of binding affinities between PFHxS and GAD isoforms, human-derived rather than mouse-derived GADs were used, as the docking relied on protein sequences predicted by AlphaFold2, and the corresponding mouse sequences are not yet available. Nevertheless, these enzymes are highly conserved across species.

This work provides evidence that gestational exposure to a human-realistic dose of PFHxS induces social deficits in offspring, with effects more pronounced in males. As a legacy PFAS listed under Annex A of the Stockholm Convention, PFHxS is widely detected in pregnant women worldwide. Together with previous epidemiological studies, our findings suggest that long-term social communication deficits may originate from in utero environmental exposures. Mechanistically, we show that PFHxS accumulates in the foetal brain and binds to the key rate-limiting enzyme GAD. This interaction is linked to altered E/I balance in neurotransmission networks. Collectively, these findings provide critical insight into how prenatal environmental exposures may lead to lasting neurodevelopmental and behavioural consequences.

## Contributors

SZ: Methodology, Validation, Formal analysis, Data curation, Writing-original draft, Writing - review & editing; CG: Conceptualisation, Methodology, Formal analysis, Data curation, Writing-original draft, Writing - review & editing, Funding acquisition; RL: Methodology, Visualisation, Writing -review & editing; JL: Conceptualisation, Methodology, Writing-review & editing. JX: Methodology, Writing - review & editing; MM: Methodology, Writing-review & editing; HL: Methodology, Writing -review & editing; D-XX: Resources, Writing - review.&. editing, Supervision, Project-administration; BW: Validation, Resources, Writing-review & editing, Supervision, Project-administration; YH: Conceptualisation, Validation, Resources, Writing-review & editing, Supervision, Project-administration, Funding acquisition. All authors have full accessed to all data generated and analysed in the study. SZ, BW and YH accessed and verified the underlying data. All authors read and approved the final version of the manuscript for submission to publication.

## Data sharing statement

The data underlying the findings reported in the current study are provided in the Supplementary Materials. Additional data are available from the corresponding author (yichao.huang@ahmu.edu.cn) upon reasonable request and subject to approval of a proposal. The RNA sequencing data generated and analysed during this study have been deposited to the China National Center for Bioinformation under accession number CRA033498, readily available from 13th January 2026.

## Declaration of interests

None declared.
