# Supplementary material for: Effects of gestational perfluorohexanesulfonic acid exposure at human realistic dose on social communication deficit in mouse offspring
Source: eBioMedicine. 2026 Feb 10;125:106160. doi: 10.1016/j.ebiom.2026.106160 (PMC12914860; doi:10.1016/j.ebiom.2026.106160)
Supplement: Tables S1–S27 [file mmc1.docx]

**SUPPORTING INFORMATION**

**For**

**Gestational Perfluorohexanesulfonic Acid Exposure at Human Realistic Dose Causes Social Communication Deficit in Mouse Offspring via Imbalance in E/I Neurotransmission**

Shengmei Zhang ^a, ‡^, Chang Gao ^a, b, ‡^, Ruonan Li ^a, ‡^, Jia Lv ^a^, Jingjing Xu ^a^, Maohua Miao ^c^, Hong Liang ^c^, De-Xiang Xu ^a, *^, Bo Wang ^a, *^, Yichao Huang ^a, b, *^

**Affiliations:**

*^a^* *School of Public Health* *and Center for Big Data and Population Health of IHM, Anhui Medical University, Hefei, 230031, China*

*^b^ Department of Pediatrics, Suzhou Hospital of Anhui Medical University, Anhui Medical University, Suzhou,* *234099, China*

*^c^ Shanghai-MOST Key Laboratory of Health and Disease Genomics, NHC Key Lab of Reproduction Regulation, Shanghai Institute for Biomedical and Pharmaceutical Technologies, Shanghai 200237, China*

^‡^ These authors contributed equally to this work.

* Address correspondence Profs **De-Xiang Xu**, **Bo Wang** and **Yichao Huang**, Department of Toxicology, School of Public Health, Anhui Medical University, 81 Meishan Rd, Hefei 230032, China. E-mails: [xudex@126.com](mailto:xudex@126.com); [wangbdev@ahmu.edu.cn](mailto:wangbdev@ahmu.edu.cn); and [yichao.huang@ahmu.edu.cn](mailto:yichao.huang@ahmu.edu.cn)

**Table of Content**

| **Figure/Table No.** | **Legend** | **Page No.** |
| --- | --- | --- |
| Table S1 | Instrument information for PFHxS measurement | S4 |
| Table S2 | Recovery tests for PFHxS measurement. | S5 |
| Table S3 | Instrument information for neurotransmitters and metabolites measurement. | S6 |
| Table S4 | Plasma concentration of PFHxS of control and exposed pregnant mice. | S7 |
| Table S5 | Relative brain weight of GD 18 fetus and PNW 4/10 offsprings. | S8 |
| Table S6 | PFHxS concentration in the mPFC of GD 18 fetus and PNW 4/10 offsprings. | S10 |
| Table S7 | Sniffing time recorded for male offspring at PNW 4. | S11 |
| Table S8 | Social preference and social novelty preference indices for male offspring at PNW 4. | S12 |
| Table S9 | Sniffing time recorded for female offspring at PNW 4. | S13 |
| Table S10 | Social preference and social novelty preference indices for female offspring at PNW 4. | S14 |
| Table S11 | Sniffing time recorded for male offspring at PNW 10. | S15 |
| Table S12 | Social preference and social novelty preference indices for male offspring at PNW 10. | S16 |
| Table S13 | Sniffing time recorded for female offspring at PNW 10. | S17 |
| Table S14 | Social preference and social novelty preference indices for female offspring at PNW 10. | S18 |
| Table S15 | Concentration of neurotransmitters and related metabolites in mPFC of male offspring of PFHxS-High group at PNW 10 (μg/g). | S19 |
| Table S16 | Concentration of neurotransmitters and related metabolites in mPFC of female offspring of PFHxS-High group at PNW 10 (μg/g). | S20 |
| Table S17 | Differentially expressed genes in mPFC of GD 18 male fetus between control and PFHxS-high groups. | S21 |
| Table S18 | Differentially expressed genes in mPFC of PNW 4 male offsprings between control and PFHxS-high groups. | S22 |
| Table S19 | Reactome annotation for differentially expressed genes in mPFC of GD 18 male fetus between control and PFHxS-high groups. | S23 |
| Table S20 | Reactome annotation for differentially expressed genes in mPFC of PNW 4 male offspring between control and PFHxS-high groups. | S24 |
| Table S21 | GO annotation for differentially expressed genes in mPFC of GD 18 male fetus between control and PFHxS-High groups. | S25 |
| Table S22 | GO annotation for differentially expressed genes in mPFC of PNW 4 male offspring between control and PFHxS-High groups. | S26 |
| Table S23 | Gene Set Variation Analysis based on differentially expressed genes in mPFC of GD18 male offspring between control and PFHxS-high groups. | S27 |
| Table S24 | Gene Set Variation Analysis based on differentially expressed genes in mPFC of PNW 4 male offspring between control and PFHxS-high groups. | S28 |
| Table S25 | Quantification of GABAergic neurons in mPFC of PNW 4 and 10 offsprings. | S29 |
| Table S26 | Expression of glutamate decarboxylase in mPFC of GD 18 fetal mice measured by Western blotting. | S30 |
| Table S27 | GABA-Glutamate ratio measured in mPFC of PNW 10 offspring mice. | S31 |

**Table S1.** Instrument information for PFHxS measurement

| **Variance** | **Parameters** | | |
| --- | --- | --- | --- |
| **LC – MS/MS Instrument** | AB SCIEX Triple Quad 5500 | | |
| **Chromatographic Column** | Zorbax Eclipse Plus C18 | | |
|  | (50 mm × 2.1 mm, 1.8 μm) | | |
| **Column Temperature** | 45 °C | | |
| **Injection Volume** | 10 µL | | |
| **Autosampler Temperature** | 4 °C | | |
| **Flow Rate** | 0.2 mL/min | | |
| **Mobile Phase A** | water, acetonitrile, and isopropyl alcohol (50:30:20, v/v/v) | | |
| **Mobile Phase B** | water, acetonitrile, and isopropyl alcohol (1:9:90, v/v/v) | | |
|  | Time | A% | B% |
|  | 0 | 80 | 20 |
|  | 3 | 20 | 80 |
| **Gradient Conditions** | 8 | 5 | 95 |
|  | 11 | 5 | 95 |
|  | 11.1 | 80 | 20 |
|  | 15 | 80 | 20 |
| **Ionization Methods** | Electrospray Ionization (ESI) | | |

**Table S2.** Recovery tests for PFHxS measurement.

| **Matrix** | **Blank spiking**  **(10 ng/mL)** | **mPFC tissue spiking (10 ng/mL)** | **mPFC tissue spiking (10 ng/mL)** |
| --- | --- | --- | --- |
| Target Analyte | PFHxS | | PFOA |
| Replicate 1 | 84.70 | 91.15 | 86.12 |
| Replicate 2 | 89.77 | 87.61 | 93.81 |
| Replicate 3 | 96.51 | 85.97 | 94.99 |
| Replicate 4 | 89.93 | 72.89 | 70.81 |
| Mean | 90.23 | 84.41 | 86.43 |
| SD | 0.05 | 0.08 | 0.11 |

**Table S3.** Instrument information for neurotransmitters and metabolites measurement.

| **Variance** | **Parameters** | | |
| --- | --- | --- | --- |
| **LC – MS/MS Instrument** | AB SCIEX Triple Quad 5500 | | |
| **Chromatographic Column** | ACQUITYTM Premier HSS T3 column | | |
|  | (1.8 μm, 2.1×150 mm,) | | |
| **Column Temperature** | 45 °C | | |
| **Injection Volume** | 10 µl | | |
| **Autosampler Temperature** | 4 °C | | |
| **Flow Rate** | 0.2 mL/min | | |
| **Mobile Phase A** | 0.1% formic acid in aqueous solution | | |
| **Mobile Phase B** | 0.1% formic acid in acetonitrile solution | | |
| **Gradient Conditions** | Time | A% | B% |
|  | 0 | 98 | 2 |
|  | 1 | 96 | 4 |
|  | 1.5 | 95 | 5 |
|  | 2.8 | 92 | 8 |
|  | 5 | 90 | 10 |
|  | 6 | 70 | 30 |
|  | 9 | 70 | 30 |
|  | 9.3 | 90 | 10 |
|  | 12.5 | 90 | 10 |
|  | 12.6 | 100 | 0 |
|  | 19 | 100 | 0 |
|  | 19.01 | 100 | 0 |
| **Internal standard** | *L-*Phenyl-d5-alanine-2,3,3-d3, TRC | | |

**Table S4.** Plasma concentration of PFHxS of control and exposed pregnant mice.

|  | Control | PFHxS-Low | PFHxS-High |
| --- | --- | --- | --- |
| Replicates | 0.058 | 1.033 | 4.743 |
|  | 0.059 | 1.227 | 4.949 |
|  | 0.063 | 2.260 | 5.164 |
|  | 0.072 | 2.337 | 5.383 |
|  | 0.153 | 3.288 | 5.463 |
| Mean | 0.081 | 2.029 | 5.140 |
| SEM | 0.018 | 0.410 | 0.134 |
| *p*_overall_ | <0.0001 | | |
| *p*_Control-Low_ | 0.0004 | | |
| *p*_Control-High_ | <0.0001 | | |
| *p*_Low-High_ | <0.0001 | | |

**Table S5**. Relative brain weight of GD 18 fetus and PNW 4/10 offsprings.

|  | **Male** | | | **Female** | | |
| --- | --- | --- | --- | --- | --- | --- |
|  | **Control** | **PFHxS-Low** | **PFHxS-High** | **Control** | **PFHxS-Low** | **PFHxS-High** |
| **GD 18** | 0.056 | 0.056 | 0.063 | 0.040 | 0.050 | 0.060 |
|  | 0.056 | 0.063 | 0.058 | 0.059 | 0.056 | 0.061 |
|  | 0.063 | 0.056 | 0.061 | 0.057 | 0.053 | 0.061 |
|  | 0.060 | 0.054 | 0.060 | 0.060 | 0.058 | 0.061 |
|  | 0.061 | 0.050 | 0.065 | 0.060 | 0.055 | 0.065 |
|  | 0.047 | 0.066 | 0.055 | 0.048 | 0.060 | 0.059 |
|  | 0.055 | 0.059 | 0.056 | 0.057 | 0.058 | 0.059 |
|  | 0.059 | 0.066 | 0.059 | 0.057 | 0.065 | 0.061 |
|  | 0.061 | 0.061 | 0.062 | 0.061 | 0.065 | 0.066 |
|  | 0.057 | 0.056 | 0.058 | 0.062 | 0.061 | 0.057 |
|  | 0.056 | 0.066 | 0.057 | 0.059 | 0.064 | 0.060 |
|  |  | 0.061 | 0.062 |  | 0.060 | 0.061 |
|  |  | 0.054 | 0.057 |  | 0.056 | 0.060 |
| *p* _Overall_ | 0.461 | | | 0.081 | | |
| *p* _Control vs. Low_ | 0.596 | | | 0.504 | | |
| *p* _Control vs. High_ | 0.463 | | | 0.066 | | |
| *p* _Low vs. High_ | 0.972 | | | 0.433 | | |
| **PNW 4** | 0.014 | 0.014 | 0.015 | 0.018 | 0.021 | 0.016 |
|  | 0.014 | 0.015 | 0.014 | 0.020 | 0.019 | 0.016 |
|  | 0.014 | 0.017 | 0.015 | 0.020 | 0.018 | 0.017 |
|  | 0.014 | 0.012 | 0.015 | 0.017 | 0.019 | 0.019 |
|  | 0.014 | 0.015 | 0.014 | 0.016 | 0.018 | 0.020 |
|  | 0.015 | 0.014 | 0.015 | 0.018 | 0.018 | 0.016 |
|  | 0.015 | 0.015 | 0.016 | 0.019 | 0.017 | 0.017 |
|  | 0.014 | 0.015 | 0.015 | 0.018 | 0.016 | 0.018 |
|  | 0.016 | 0.015 | 0.015 | 0.017 | 0.019 | 0.018 |
|  |  |  | 0.015 |  |  |  |
| *p* _Overall_ | 0.566 | | | 0.212 | | |
| *p* _Control vs. Low_ | 0.866 | | | 0.944 | | |
| *p* _Control vs. High_ | 0.536 | | | 0.372 | | |
| *p* _Low vs. High_ | 0.846 | | | 0.224 | | |
| **PNW 10** | 0.014 | 0.013 | 0.012 | 0.016 | 0.018 | 0.015 |
|  | 0.013 | 0.012 | 0.014 | 0.016 | 0.017 | 0.017 |
|  | 0.012 | 0.012 | 0.012 | 0.016 | 0.017 | 0.016 |
|  | 0.012 | 0.013 | 0.012 | 0.016 | 0.017 | 0.014 |
|  | 0.012 | 0.011 | 0.013 | 0.017 | 0.016 | 0.016 |
|  | 0.014 | 0.013 | 0.014 | 0.017 | 0.019 | 0.015 |
|  | 0.013 | 0.014 | 0.014 | 0.015 | 0.014 | 0.016 |
|  | 0.014 | 0.013 | 0.011 | 0.016 | 0.016 | 0.014 |
|  | 0.012 | 0.015 | 0.012 | 0.017 | 0.016 | 0.018 |
|  | 0.012 | 0.013 | 0.013 | 0.018 | 0.015 | 0.017 |
| *p* _Overall_ | 0.910 | | | 0.397 | | |
| *p* _Control vs. Low_ | 0.974 | | | 0.982 | | |
| *p* _Control vs. High_ | 0.974 | | | 0.525 | | |
| *p* _Low vs. High_ | 0.902 | | | 0.419 | | |

**Table S6.** PFHxS concentration in the mPFC of GD 18 fetus and PNW 4/10 offsprings.

|  | **GD 18** | | | **PNW 4** | | | **PNW 10** | | |
| --- | --- | --- | --- | --- | --- | --- | --- | --- | --- |
|  | **Control** | **PFHxS-Low** | **PFHxS-High** | **Control** | **PFHxS-Low** | **PFHxS-High** | **Control** | **PFHxS-Low** | **PFHxS-High** |
| Replicates | 0.209 | 10.581 | 75.805 | 1.618 | 1.050 | 3.263 | 0.883 | 1.418 | 1.635 |
|  | 0.215 | 12.095 | 52.943 | 1.226 | 1.545 | 3.335 | 0.524 | 0.862 | 1.397 |
|  | 0.310 | 11.494 | 68.635 | 0.881 | 1.500 | 3.979 | 0.221 | 1.470 | 0.708 |
|  | 1.787 | 8.764 | 68.775 | 0.680 | 2.510 | 2.801 | 0.580 | 0.640 | 0.923 |
|  | 0.281 | 12.585 | 74.504 | 0.305 | 1.586 | 1.676 | 0.326 | 1.061 | 1.089 |
| Mean | 0.560 | 11.10 | 68.13 | 0.942 | 1.64 | 3.01 | 0.507 | 1.09 | 1.15 |
| SEM | 0.307 | 0.674 | 4.07 | 0.225 | 0.238 | 0.383 | 0.114 | 0.159 | 0.165 |
| *p* _Overall_ | <0.0001 | | | 0.0010 | | | 0.0176 | | |
| *p* _Control vs. Low_ | 0.0222 | | | 0.2483 | | | 0.0406 | | |
| *p* _Control vs. High_ | <0.0001 | | | 0.0008 | | | 0.0243 | | |
| *p* _Low vs. High_ | <0.0001 | | | 0.0152 | | | 0.9556 | | |

**Table S7.** Sniffing time recorded for male offspring at PNW 4.

| **Group** | **Sniffing time (s)** | | | |
| --- | --- | --- | --- | --- |
|  | **S1** | **E** | **S1** | **S2** |
| Control | 107.25 | 77.56 | 17.94 | 40.12 |
|  | 144.44 | 101.06 | 12.06 | 160.25 |
|  | 194.00 | 156.06 | 108.06 | 103.56 |
|  | 159.19 | 36.31 | 62.00 | 219.50 |
|  | 175.31 | 59.69 | 61.44 | 82.19 |
|  | 163.31 | 66.12 | 41.00 | 116.62 |
|  | 195.19 | 22.37 | 49.87 | 75.87 |
|  | 84.19 | 119.12 | 33.81 | 55.37 |
|  | 143.25 | 72.31 | 117.56 | 81.69 |
|  | 162.94 | 60.31 | 77.69 | 126.38 |
| Mean | 152.9 | 77.09 | 58.14 | 106.2 |
| SEM | 11.15 | 12.46 | 11.12 | 16.78 |
| *p* | 0.0003 | | 0.028 | |
| PFHxS-Low | 138.37 | 86.00 | 44.81 | 89.25 |
|  | 112.81 | 64.31 | 35.50 | 205.19 |
|  | 55.37 | 115.87 | 30.44 | 80.94 |
|  | 122.25 | 65.06 | 79.87 | 138.31 |
|  | 178.44 | 49.69 | 97.25 | 96.12 |
|  | 103.19 | 73.31 | 40.50 | 63.31 |
|  | 169.12 | 78.56 | 103.44 | 109.75 |
|  | 216.31 | 51.44 | 95.38 | 146.87 |
|  | 149.50 | 69.19 | 68.94 | 73.56 |
|  | 97.37 | 107.88 | 21.12 | 84.25 |
| Mean | 134.3 | 76.13 | 61.73 | 108.8 |
| SEM | 14.64 | 6.93 | 9.76 | 13.68 |
| *p* | 0.002 | | 0.012 | |
| PFHxS-High | 113.37 | 58.75 | 101.87 | 86.94 |
|  | 204.00 | 53.19 | 89.56 | 80.12 |
|  | 318.06 | 54.50 | 93.37 | 76.12 |
|  | 129.19 | 49.50 | 37.19 | 49.25 |
|  | 80.69 | 29.12 | 94.31 | 36.12 |
|  | 147.37 | 62.62 | 110.37 | 87.19 |
|  | 145.44 | 37.75 | 105 | 118.94 |
|  | 99.25 | 49.19 | 67.87 | 55.88 |
|  | 184.94 | 83.37 | 210.5 | 36.12 |
| Mean | 158.0 | 53.11 | 101.1 | 69.63 |
| SEM | 23.86 | 5.11 | 15.59 | 9.16 |
| *p* | 0.0006 | | 0.101 | |

**Table S8.** Social preference and social novelty preference indices for male offspring at PNW 4.

|  | **Social preference index** | | | **Social novelty preference index** | | |
| --- | --- | --- | --- | --- | --- | --- |
|  | **Control** | **PFHxS - Low** | **PFHxS - High** | **Control** | **PFHxS - Low** | **PFHxS - High** |
| Replicates | 0.161 | 0.233 | 0.317 | 0.382 | 0.331 | -0.079 |
|  | 0.177 | 0.274 | 0.586 | 0.860 | 0.705 | -0.056 |
|  | 0.108 | -0.353 | 0.707 | -0.021 | 0.453 | -0.102 |
|  | 0.629 | 0.305 | 0.446 | 0.560 | 0.268 | 0.140 |
|  | 0.492 | 0.564 | 0.470 | -0.146 | -0.006 | -0.446 |
|  | 0.424 | 0.169 | 0.404 | 0.480 | 0.220 | -0.117 |
|  | 0.794 | 0.366 | 0.588 | 0.207 | 0.030 | 0.062 |
|  | -0.172 | 0.616 | 0.337 | 0.242 | 0.213 | -0.097 |
|  | 0.329 | 0.367 | -0.997 | -0.180 | 0.032 | -0.707 |
|  | 0.460 | -0.051 |  | 0.239 | 0.599 |  |
| Mean | 0.340 | 0.249 | 0.318 | 0.262 | 0.285 | -0.156 |
| SEM | 0.089 | 0.090 | 0.170 | 0.103 | 0.077 | 0.087 |
| *p* _Overall_ | 0.8477 | | | 0.0030 | | |
| *p* _Control vs. Low_ | 0.8447 | | | 0.9828 | | |
| *p* _Control vs. High_ | 0.9901 | | | 0.0085 | | |
| *p* _Low vs. High_ | 0.9133 | | | 0.0056 | | |

**Table S9.** Sniffing time recorded for female offspring at PNW 4.

| Group | **Sniffing time (s)** | | | |
| --- | --- | --- | --- | --- |
|  | **S1** | **E** | **S1** | **S2** |
| Control | 77.69 | 70.69 | 11.37 | 53.81 |
|  | 114.75 | 123.31 | 51.87 | 194.94 |
|  | 127.75 | 90.06 | 92.69 | 121.19 |
|  | 42.27 | 102 | 73.5 | 148.44 |
|  | 201.62 | 83.69 | 122.56 | 190 |
|  | 106.37 | 90.75 | 86.69 | 43.25 |
|  | 135.06 | 87.06 | 52.81 | 170.37 |
|  | 73.19 | 81.25 | 17.87 | 63.37 |
|  | 244.44 | 18.62 | 150.62 | 124.19 |
|  | 117.5 | 34.37 | 70.72 | 98.22 |
| Mean | 124 | 78.2 | 73.1 | 121 |
| SEM | 19.0 | 9.74 | 13.6 | 17.6 |
| *p* | 0.046 | | 0.046 | |
| PFHxS-Low | 156.62 | 51.19 | 137.44 | 75.12 |
|  | 92.87 | 124.5 | 132.62 | 34.19 |
|  | 172.37 | 26.31 | 79.37 | 143.31 |
|  | 75.06 | 103.37 | 131.94 | 39.75 |
|  | 102.06 | 78.62 | 27.31 | 81.44 |
|  | 129.44 | 49.44 | 188.94 | 110.19 |
|  | 194.31 | 45 | 39.51 | 141.81 |
|  | 109.94 | 57.06 | 92.87 | 31.81 |
|  | 92.38 | 64.75 | 95.56 | 134.56 |
|  | 162.25 | 152.06 | 64 | 102.62 |
| Mean | 129 | 75.2 | 99.0 | 89.5 |
| SEM | 12.8 | 12.5 | 15.7 | 13.9 |
| *p* | 0.008 | | 0.657 | |
| PFHxS-High | 132.06 | 96.69 | 37.81 | 72.94 |
|  | 71.12 | 64.19 | 88.06 | 40.19 |
|  | 184.81 | 96.37 | 118.5 | 91.12 |
|  | 122.94 | 169.19 | 209.5 | 51.87 |
|  | 120.37 | 185.31 | 45.31 | 187.62 |
|  | 107 | 99 | 59.56 | 25.56 |
|  | 175.81 | 97.62 | 75.12 | 88.25 |
|  | 74.5 | 119.5 | 91.12 | 170.31 |
|  | 93.62 | 64.37 | 58.31 | 108.75 |
|  | 219 | 53.44 | 132.87 | 120.62 |
| Mean | 130 | 105 | 91.6 | 95.7 |
| SEM | 15.5 | 13.7 | 16.3 | 16.8 |
| *p* | 0.233 | | 0.862 | |

**Table S10.** Social preference and social novelty preference indices for female offspring at PNW 4.

|  | **Social preference index** | | | **Social novelty preference index** | | |
| --- | --- | --- | --- | --- | --- | --- |
|  | **Control** | **PFHxS - Low** | **PFHxS - High** | **Control** | **PFHxS - Low** | **PFHxS - High** |
| Replicates | 0.047 | 0.507 | 0.155 | 0.651 | -0.293 | 0.317 |
|  | -0.036 | -0.146 | 0.051 | 0.580 | -0.590 | -0.373 |
|  | 0.173 | 0.735 | 0.315 | 0.133 | 0.287 | -0.131 |
|  | -0.414 | -0.159 | -0.158 | 0.338 | -0.537 | -0.603 |
|  | 0.413 | 0.130 | -0.212 | 0.216 | 0.498 | 0.611 |
|  | 0.079 | 0.447 | 0.039 | -0.334 | -0.263 | -0.399 |
|  | 0.216 | 0.624 | 0.286 | 0.527 | 0.564 | 0.080 |
|  | -0.052 | 0.317 | -0.232 | 0.560 | -0.490 | 0.303 |
|  | 0.858 | 0.176 | 0.185 | -0.096 | 0.169 | 0.302 |
|  | 0.547 | 0.032 | 0.608 | 0.230 | 0.232 | -0.048 |
| Mean | 0.183 | 0.266 | 0.104 | 0.281 | -0.042 | 0.006 |
| SEM | 0.112 | 0.099 | 0.084 | 0.101 | 0.139 | 0.122 |
| *p* _Overall_ | 0.516 | | | 0.149 | | |
| *p* _Control vs. Low_ | 0.824 | | | 0.166 | | |
| *p* _Control vs. High_ | 0.838 | | | 0.265 | | |
| *p* _Low vs. High_ | 0.484 | | | 0.958 | | |

**Table S11.** Sniffing time recorded for male offspring at PNW 10.

| **Group** | **Sniffing time (s)** | | | |
| --- | --- | --- | --- | --- |
|  | **S1** | **E** | **S1** | **S2** |
| Control | 207.44 | 70.19 | 127.50 | 194.75 |
|  | 159.31 | 75.87 | 38.00 | 97.69 |
|  | 87.75 | 85.00 | 48.00 | 173.44 |
|  | 262.81 | 53.44 | 93.06 | 163.62 |
|  | 146.44 | 28.56 | 36.94 | 105.19 |
|  | 168.87 | 59.31 | 101.75 | 120.69 |
|  | 147.00 | 94.00 | 63.50 | 138.62 |
|  | 109.87 | 63.81 | 31.25 | 107.00 |
|  | 140.69 | 40.19 | 54.50 | 80.50 |
|  | 256.69 | 40.50 | 50.06 | 122.75 |
| Mean | 169 | 61.1 | 64.5 | 130 |
| SEM | 18.2 | 6.62 | 10.2 | 11.6 |
| *p* | <0.0001 | | 0.0005 | |
| PFHxS-Low | 106.50 | 62.87 | 47.69 | 121.94 |
|  | 113.94 | 53.94 | 38.25 | 72.37 |
|  | 73.06 | 53.19 | 87.31 | 19.69 |
|  | 66.37 | 76.81 | 90.31 | 105.75 |
|  | 137.00 | 69.69 | 116.12 | 55.69 |
|  | 140.56 | 43.69 | 31.31 | 127.94 |
|  | 180.44 | 64.00 | 47.87 | 108.62 |
|  | 109.94 | 41.94 | 40.12 | 92.31 |
|  | 214.94 | 37.19 | 30.75 | 106.94 |
|  | 164.81 | 86.94 | 185.06 | 129.44 |
| Mean | 131 | 59.0 | 71.5 | 94.1 |
| SEM | 14.8 | 5.07 | 15.6 | 11.1 |
| *p* | 0.0002 | | 0.255 | |
| PFHxS-High | 218.75 | 41.75 | 98.38 | 172.75 |
|  | 52.06 | 83.44 | 50.94 | 26.75 |
|  | 148.50 | 106.13 | 132.81 | 60.56 |
|  | 186.37 | 59.25 | 170.69 | 127.06 |
|  | 144.75 | 86.75 | 90.50 | 110.56 |
|  | 106.94 | 56.00 | 70.25 | 85.37 |
|  | 103.37 | 38.94 | 79.12 | 118.69 |
|  | 167.69 | 38.44 | 75.25 | 81.87 |
|  | 16.06 | 34.12 | 44.37 | 78.69 |
|  | 222.94 | 33.37 | 59.00 | 18.31 |
| Mean | 137 | 57.8 | 87.1 | 88.1 |
| SEM | 21.5 | 8.15 | 12.3 | 14.8 |
| *p* | 0.003 | | 0.962 | |

**Table S12.** Social preference and social novelty preference indices for male offspring at PNW 10.

|  | **Social preference index** | | | **Social novelty preference index** | | |
| --- | --- | --- | --- | --- | --- | --- |
|  | **Control** | **PFHxS - Low** | **PFHxS - High** | **Control** | **PFHxS - Low** | **PFHxS - High** |
| Replicates | 0.494 | 0.258 | 0.679 | 0.209 | 0.438 | 0.274 |
|  | 0.355 | 0.357 | -0.232 | 0.440 | 0.308 | -0.311 |
|  | 0.016 | 0.157 | 0.166 | 0.566 | -0.632 | -0.374 |
|  | 0.662 | -0.073 | 0.518 | 0.275 | 0.079 | -0.147 |
|  | 0.674 | 0.326 | 0.251 | 0.480 | -0.352 | 0.100 |
|  | 0.480 | 0.526 | 0.313 | 0.085 | 0.607 | 0.097 |
|  | 0.220 | 0.476 | 0.453 | 0.372 | 0.388 | 0.200 |
|  | 0.265 | 0.448 | 0.627 | 0.548 | 0.394 | 0.042 |
|  | 0.556 | 0.705 | -0.360 | 0.193 | 0.553 | 0.279 |
|  | 0.727 | 0.309 | 0.740 | 0.421 | -0.177 | -0.526 |
| Mean | 0.445 | 0.349 | 0.316 | 0.359 | 0.161 | -0.037 |
| SEM | 0.0723 | 0.0674 | 0.118 | 0.051 | 0.132 | 0.090 |
| *p* _Overall_ | 0.571 | | | 0.027 | | |
| *p* _Control vs. Low_ | 0.728 | | | 0.333 | | |
| *p* _Control vs. High_ | 0.565 | | | 0.020 | | |
| *p* _Low vs. High_ | 0.962 | | | 0.337 | | |

**Table S13.** Sniffing time recorded for female offspring at PNW 10.

| **Group** | **Sniffing time (s)** | | | |
| --- | --- | --- | --- | --- |
|  | **S1** | **E** | **S1** | **S2** |
| Control | 114.56 | 133.94 | 28.25 | 124.06 |
|  | 111.44 | 114.75 | 58.21 | 115.81 |
|  | 100.19 | 42.37 | 59.31 | 79 |
|  | 160.94 | 67.94 | 86.62 | 110.44 |
|  | 103.62 | 155.75 | 32 | 123.38 |
|  | 98.87 | 77.19 | 62.19 | 76.06 |
|  | 169.31 | 31.87 | 74.37 | 106.87 |
|  | 88.56 | 60.75 | 53.37 | 80.69 |
|  | 77.37 | 53.37 | 48.87 | 62.69 |
|  | 168.69 | 58.25 | 48.06 | 108.12 |
| Mean | 119 | 79.6 | 55.1 | 98.7 |
| SEM | 10.8 | 13.0 | 5.57 | 6.96 |
| *p* | 0.031 | | 0.0001 | |
| PFHxS-Low | 159.62 | 73.75 | 96.87 | 60.94 |
|  | 195.37 | 26.31 | 61.5 | 95.5 |
|  | 113.88 | 97.81 | 68.56 | 165.87 |
|  | 130.06 | 72.82 | 46.5 | 147.25 |
|  | 85.69 | 81.81 | 39.62 | 80.44 |
|  | 92.61 | 61.81 | 84.5 | 140.19 |
|  | 26.12 | 43.87 | 33.19 | 31.87 |
|  | 109.06 | 93.5 | 65.75 | 71.44 |
|  | 141.56 | 75.31 | 61.31 | 101.75 |
|  | 111.69 | 57.19 | 48.5 | 131.81 |
| Mean | 117 | 68.4 | 60.6 | 103 |
| SEM | 14.4 | 6.92 | 6.25 | 13.5 |
| *p* | 0.008 | | 0.011 | |
| PFHxS-High | 158.37 | 80.31 | 10.25 | 186.44 |
|  | 76.37 | 83.37 | 18.44 | 78.25 |
|  | 68.31 | 96.37 | 46.19 | 172.19 |
|  | 82.62 | 44.69 | 26.56 | 165.62 |
|  | 139.81 | 110.69 | 89.87 | 180.75 |
|  | 75.75 | 92.06 | 132 | 137.94 |
|  | 59.94 | 105.44 | 58.25 | 71.44 |
|  | 53.69 | 100.19 | 78.25 | 85.19 |
|  | 79.44 | 75.62 | 22.56 | 110.88 |
|  | 69.81 | 24.06 | 44 | 44.75 |
| Mean | 86.4 | 81.3 | 52.6 | 123 |
| SEM | 10.9 | 8.69 | 12.0 | 16.4 |
| *p* | 0.717 | | 0.003 | |

**Table S14.** Social preference and social novelty preference indices for female offspring at PNW 10.

|  | **Social preference index** | | | **Social novelty preference index** | | |
| --- | --- | --- | --- | --- | --- | --- |
|  | **Control** | **PFHxS - Low** | **PFHxS - High** | **Control** | **PFHxS - Low** | **PFHxS - High** |
| Replicates | -0.078 | 0.368 | 0.327 | 0.629 | -0.228 | 0.896 |
|  | -0.015 | 0.763 | -0.044 | 0.331 | 0.217 | 0.619 |
|  | 0.406 | 0.076 | -0.170 | 0.142 | 0.415 | 0.577 |
|  | 0.406 | 0.282 | 0.298 | 0.121 | 0.520 | 0.724 |
|  | -0.201 | 0.023 | 0.116 | 0.588 | 0.340 | 0.336 |
|  | 0.123 | 0.199 | -0.097 | 0.100 | 0.248 | 0.022 |
|  | 0.683 | -0.254 | -0.275 | 0.179 | -0.020 | 0.102 |
|  | 0.186 | 0.077 | -0.302 | 0.204 | 0.041 | 0.042 |
|  | 0.184 | 0.305 | 0.025 | 0.124 | 0.248 | 0.662 |
|  | 0.487 | 0.323 | 0.487 | 0.385 | 0.462 | 0.008 |
| Mean | 0.218 | 0.216 | 0.037 | 0.280 | 0.224 | 0.399 |
| SEM | 0.087 | 0.085 | 0.084 | 0.062 | 0.074 | 0.106 |
| *p* _Overall_ | 0.243 | | | 0.330 | | |
| *p* _Control vs. Low_ | 0.999 | | | 0.882 | | |
| *p* _Control vs. High_ | 0.306 | | | 0.577 | | |
| *p* _Low vs. High_ | 0.313 | | | 0.312 | | |

**Table S15.** Concentration of neurotransmitters and related metabolites in mPFC of male offspring of PFHxS-High group at PNW 10 (μg/g).

| **Group** | **Inhibitory** | | | | | | | | | **Excitatory** | | | | | | | | | | | |
| --- | --- | --- | --- | --- | --- | --- | --- | --- | --- | --- | --- | --- | --- | --- | --- | --- | --- | --- | --- | --- | --- |
|  | **5-HTP** | **PL** | **Gly** | **5-HT** | **Tau** | **Kyna** | **GABA** | **β-Ala** | **Trp** | **5-HTP** | **DA** | **His** | **Cys** | **HCA** | **NE** | **Tyr** | **Ser** | **Glu** | **γ-Glu-Phe** | **Gln** | **Asp** |
| Control | 0.070 | 0.132 | 0.126 | 0.367 | 1.807 | 0.203 | 86.029 | 0.415 | 19.538 | 0.070 | 2.342 | 0.162 | 0.025 | 0.406 | 2.941 | 11.029 | 1.155 | 115.5 | 0.119 | 48.004 | 41.071 |
|  | 0.093 | 0.136 | 0.118 | 0.400 | 1.555 | 0.110 | 85.819 | 0.389 | 24.160 | 0.093 | 3.036 | 0.189 | 0.042 | 0.465 | 2.962 | 12.710 | 1.492 | 125.0 | 0.209 | 56.933 | 44.118 |
|  | 0.036 | 0.129 | 0.183 | 0.370 | 1.815 | 0.157 | 76.660 | 0.389 | 17.739 | 0.036 | 2.718 | 0.203 | 0.030 | 0.308 | 2.645 | 9.409 | 1.888 | 131.7 | 0.166 | 57.676 | 33.817 |
|  | 0.042 | 0.152 | 0.154 | 0.479 | 1.957 | 0.126 | 95.435 | 0.422 | 21.957 | 0.042 | 2.946 | 0.200 | 0.076 | 0.503 | 2.554 | 15.978 | 1.717 | 133.7 | 0.239 | 57.609 | 40.543 |
|  | 0.009 | 0.212 | 0.227 | 0.709 | 2.456 | 0.154 | 108.9 | 0.440 | 27.047 | 0.009 | 4.050 | 0.285 | 0.043 | 0.344 | 4.079 | 14.211 | 2.544 | 178.4 | 0.141 | 85.088 | 57.164 |
| Mean | 0.050 | 0.152 | 0.161 | 0.465 | 1.918 | 0.150 | 90.6 | 0.411 | 22.088 | 0.050 | 3.018 | 0.208 | 0.043 | 0.405 | 3.036 | 12.667 | 1.759 | 136.9 | 0.175 | 61.062 | 43.343 |
| SEM | 0.033 | 0.035 | 0.044 | 0.144 | 0.334 | 0.035 | 12.217 | 0.022 | 3.685 | 0.033 | 0.636 | 0.046 | 0.020 | 0.081 | 0.610 | 2.581 | 0.517 | 24.3 | 0.049 | 14.037 | 8.593 |
| PFHxS-High | 0.062 | 0.102 | 0.085 | 0.386 | 1.451 | 0.093 | 65.487 | 0.347 | 18.142 | 0.062 | 2.097 | 0.177 | 0.022 | 0.316 | 1.558 | 10.354 | 1.115 | 123.0 | 0.056 | 53.451 | 29.115 |
|  | 0.088 | 0.150 | 0.107 | 0.459 | 1.455 | 0.107 | 67.453 | 0.385 | 20.256 | 0.088 | 2.183 | 0.148 | 0.052 | 0.272 | 2.576 | 12.389 | 1.642 | 123.9 | 0.039 | 51.426 | 46.706 |
|  | 0.074 | 0.136 | 0.109 | 0.326 | 1.295 | 0.092 | 76.584 | 0.409 | 16.621 | 0.074 | 1.983 | 0.179 | 0.035 | 0.474 | 2.902 | 11.111 | 1.387 | 127.6 | 0.283 | 51.607 | 38.017 |
|  | 0.049 | 0.151 | 0.123 | 0.400 | 1.606 | 0.172 | 69.523 | 0.363 | 21.053 | 0.049 | 2.252 | 0.174 | 0.023 | 0.346 | 2.634 | 11.359 | 1.479 | 126.3 | 0.029 | 56.695 | 45.435 |
|  | 0.045 | 0.140 | 0.114 | 0.382 | 1.419 | 0.116 | 71.895 | 0.349 | 20.075 | 0.045 | 2.525 | 0.295 | 0.033 | 0.327 | 2.260 | 13.445 | 1.289 | 119.5 | 0.040 | 46.499 | 47.806 |
| Mean | 0.064 | 0.136 | 0.108 | 0.391 | 1.445 | 0.116 | 70.188 | 0.371 | 19.229 | 0.064 | 2.208 | 0.195 | 0.033 | 0.347 | 2.386 | 11.732 | 1.382 | 124.1 | 0.089 | 51.936 | 41.416 |
| SEM | 0.018 | 0.020 | 0.014 | 0.048 | 0.111 | 0.033 | 4.297 | 0.026 | 1.808 | 0.018 | 0.203 | 0.057 | 0.012 | 0.076 | 0.516 | 1.203 | 0.198 | 3.150 | 0.109 | 3.704 | 7.871 |
| *p* | 0.434 | 0.388 | **0.033** | 0.304 | **0.017** | 0.156 | **0.008** | **0.031** | 0.158 | 0.434 | 0.106 | 0.702 | 0.350 | 0.275 | 0.148 | 0.484 | 0.166 | 0.276 | 0.282 | 0.197 | 0.721 |
| *q* | 0.434 | 0.434 | **0.074** | 0.391 | **0.074** | 0.237 | **0.072** | **0.074** | 0.237 | 0.579 | 0.483 | 0.721 | 0.525 | 0.483 | 0.483 | 0.581 | 0.483 | 0.483 | 0.483 | 0.483 | 0.721 |

**Table S16.** Concentration of neurotransmitters and related metabolites in mPFC of female offspring of PFHxS-High group at PNW 10 (μg/g).

| **Group** | **Inhibitory** | | | | | | | | | **Excitatory** | | | | | | | | | | | |
| --- | --- | --- | --- | --- | --- | --- | --- | --- | --- | --- | --- | --- | --- | --- | --- | --- | --- | --- | --- | --- | --- |
|  | **5-HTP** | **PL** | **Gly** | **5-HT** | **Tau** | **Kyna** | **GABA** | **β-Ala** | **Trp** | **5-HTP** | **DA** | **His** | **Cys** | **HCA** | **NE** | **Tyr** | **Ser** | **Glu** | **γ-Glu-Phe** | **Gln** | **Asp** |
| Control | 0.111 | 0.040 | 0.157 | 0.388 | 0.901 | 0.060 | 38.996 | 0.232 | 10.960 | 0.111 | 3.674 | 0.012 | 0.006 | 0.060 | 0.110 | 17.343 | 1.796 | 31.812 | 0.122 | 41.869 | 11.452 |
|  | 0.188 | 0.053 | 0.142 | 0.445 | 1.013 | 0.050 | 36.126 | 0.210 | 9.838 | 0.188 | 3.119 | 0.018 | 0.019 | 0.058 | 0.132 | 15.321 | 2.161 | 36.126 | 0.131 | 43.545 | 15.321 |
|  | 0.185 | 0.030 | 0.149 | 0.294 | 1.002 | 0.053 | 36.657 | 0.268 | 11.730 | 0.185 | 3.198 | 0.021 | 0.007 | 0.054 | 0.090 | 17.875 | 2.070 | 39.450 | 0.162 | 48.178 | 16.408 |
|  | 0.105 | 0.060 | 0.181 | 0.312 | 0.687 | 0.035 | 33.170 | 0.215 | 10.693 | 0.105 | 3.230 | 0.026 | 0.008 | 0.063 | 0.105 | 18.178 | 1.892 | 33.388 | 0.134 | 42.553 | 14.424 |
|  | 0.162 | 0.041 | 0.163 | 0.419 | 0.902 | 0.032 | 32.193 | 0.251 | 8.714 | 0.162 | 4.524 | 0.018 | 0.021 | 0.069 | 0.116 | 12.683 | 1.635 | 33.858 | 0.093 | 46.625 | 14.209 |
| Mean | 0.150 | 0.045 | 0.159 | 0.372 | 0.901 | 0.046 | 35.428 | 0.235 | 10.387 | 0.150 | 3.549 | 0.019 | 0.012 | 0.061 | 0.111 | 16.280 | 1.911 | 34.927 | 0.128 | 44.554 | 14.363 |
| SEM | 0.040 | 0.012 | 0.015 | 0.066 | 0.131 | 0.012 | 2.752 | 0.024 | 1.154 | 0.040 | 0.586 | 0.005 | 0.007 | 0.006 | 0.015 | 2.298 | 0.211 | 2.962 | 0.025 | 2.722 | 1.844 |
| PFHxS-High | 0.202 | 0.053 | 0.141 | 0.321 | 1.246 | 0.054 | 30.531 | 0.244 | 8.820 | 0.202 | 3.652 | 0.031 | 0.010 | 0.069 | 0.104 | 13.042 | 1.907 | 33.585 | 0.142 | 44.478 | 16.170 |
|  | 0.226 | 0.042 | 0.170 | 0.309 | 1.459 | 0.040 | 32.096 | 0.292 | 9.703 | 0.226 | 2.822 | 0.024 | 0.029 | 0.064 | 0.139 | 13.015 | 2.300 | 37.647 | 0.116 | 46.511 | 16.514 |
|  | 0.191 | 0.044 | 0.153 | 0.355 | 1.062 | 0.069 | 34.541 | 0.219 | 11.725 | 0.191 | 3.613 | 0.019 | 0.009 | 0.067 | 0.151 | 17.714 | 1.765 | 37.076 | 0.158 | 47.216 | 15.844 |
|  | 0.116 | 0.061 | 0.164 | 0.204 | 0.903 | 0.043 | 35.903 | 0.200 | 10.979 | 0.116 | 2.286 | 0.019 | 0.007 | 0.060 | 0.086 | 16.327 | 1.670 | 29.667 | 0.135 | 35.336 | 13.927 |
|  | 0.254 | 0.046 | 0.167 | 0.388 | 1.129 | 0.039 | 34.307 | 0.198 | 9.143 | 0.254 | 2.870 | 0.023 | 0.017 | 0.057 | 0.123 | 15.060 | 1.848 | 38.149 | 0.153 | 48.791 | 17.864 |
| Mean | 0.197 | 0.048 | 0.164 | 0.314 | 1.138 | 0.048 | 34.212 | 0.227 | 10.388 | 0.197 | 2.898 | 0.021 | 0.016 | 0.062 | 0.125 | 15.529 | 1.896 | 35.635 | 0.140 | 44.463 | 16.037 |
| SEM | 0.052 | 0.008 | 0.012 | 0.069 | 0.208 | 0.013 | 2.138 | 0.039 | 1.237 | 0.052 | 0.580 | 0.005 | 0.009 | 0.005 | 0.026 | 2.055 | 0.242 | 3.585 | 0.017 | 5.334 | 1.421 |
| *p* | 0.140 | 0.496 | 0.965 | 0.226 | 0.046 | 0.687 | 0.246 | 0.827 | 0.690 | 0.140 | 0.212 | 0.194 | 0.673 | 0.459 | 0.481 | 0.392 | 0.932 | 0.890 | 0.376 | 0.975 | 0.141 |
| *q* | 0.554 | 0.887 | 0.965 | 0.554 | 0.414 | 0.887 | 0.554 | 0.930 | 0.887 | 0.636 | 0.636 | 0.636 | 0.897 | 0.722 | 0.722 | 0.722 | 0.975 | 0.975 | 0.722 | 0.975 | 0.636 |

**Table S17.** Differentially expressed genes in mPFC of GD 18 male fetus between control and PFHxS-high groups.

**Table S18.** Differentially expressed genes in mPFC of PNW 4 male offsprings between control and PFHxS-high groups.

**Table S19.** Reactome annotation for differentially expressed genes in mPFC of GD 18 male fetus between control and PFHxS-high groups.

**Table S20.** Reactome annotation for differentially expressed genes in mPFC of PNW 4 male offspring between control and PFHxS-high groups.

**Table S21.** GO annotation for differentially expressed genes in mPFC of GD 18 male fetus between control and PFHxS-High groups.

| **ID** | **Term** | ***p*** | ***q*** | **all** | **gene** |
| --- | --- | --- | --- | --- | --- |
| GO:0030154 | cell differentiation | <0.0001 | <0.0001 | 2184 | 73 |
| GO:0030335 | positive regulation of cell migration | <0.0001 | <0.0001 | 480 | 27 |
| GO:0030336 | negative regulation of cell migration | <0.0001 | <0.0001 | 246 | 15 |
| GO:0043025 | neuronal cell body | <0.0001 | 0.0002 | 598 | 22 |
| GO:0007272 | ensheathment of neurons | <0.0001 | 0.0003 | 103 | 9 |
| GO:0010975 | regulation of neuron projection development | <0.0001 | 0.0004 | 486 | 19 |
| GO:0043005 | neuron projection | <0.0001 | 0.0009 | 1317 | 34 |
| GO:0042552 | myelination | 0.0002 | 0.0083 | 100 | 7 |
| GO:2000179 | positive regulation of neural precursor cell proliferation | 0.0002 | 0.0106 | 48 | 5 |
| GO:0050769 | positive regulation of neurogenesis | 0.0006 | 0.0241 | 247 | 10 |
| GO:0021675 | nerve development | 0.0011 | 0.0375 | 40 | 4 |

**Table S22.** GO annotation for differentially expressed genes in mPFC of PNW 4 male offspring between control and PFHxS-high groups.

| **ID** | **Term** | ***p*** | ***q*** | **all** | **up_gene** |
| --- | --- | --- | --- | --- | --- |
| GO:1903530 | regulation of secretion by cell | <0.0001 | 0.0005 | 610 | 22 |
| GO:0098810 | neurotransmitter reuptake | <0.0001 | 0.0008 | 12 | 4 |
| GO:0050767 | regulation of neurogenesis | <0.0001 | 0.0016 | 393 | 16 |
| GO:0099177 | regulation of trans-synaptic signaling | 0.0001 | 0.0056 | 596 | 19 |
| GO:0001505 | obsolete regulation of neurotransmitter levels | 0.0001 | 0.0075 | 162 | 9 |
| GO:0098982 | GABA-ergic synapse | 0.0002 | 0.0078 | 99 | 7 |
| GO:0050807 | regulation of synapse organization | 0.0003 | 0.0144 | 220 | 10 |
| GO:0051966 | regulation of synaptic transmission, glutamatergic | 0.0006 | 0.0255 | 58 | 5 |
| GO:0060074 | synapse maturation | 0.0006 | 0.0278 | 15 | 3 |
| GO:0099536 | synaptic signaling | 0.0007 | 0.0280 | 289 | 11 |
| GO:0050768 | negative regulation of neurogenesis | 0.0010 | 0.0381 | 133 | 7 |
| GO:0006836 | neurotransmitter transport | 0.0013 | 0.0460 | 69 | 5 |

**Table S23.** Gene Set Variation Analysis based on differentially expressed genes in mPFC of GD 18 male offspring between control and PFHxS-high groups.

**Table S24.** Gene Set Variation Analysis based on differentially expressed genes in mPFC of PNW 4 male offspring between control and PFHxS-high groups.

**Table S25.** Quantification of GABAergic neurons in mPFC of PNW 4 and 10 offsprings.

| **Timepoint** |  | **Cg1** | | | **PrL** | | | **IL** | | |
| --- | --- | --- | --- | --- | --- | --- | --- | --- | --- | --- |
|  |  | **Control** | **PFHxS - Low** | **PFHxS - High** | **Control** | **PFHxS - Low** | **PFHxS - High** | **Control** | **PFHxS - Low** | **PFHxS - High** |
| **PNW 4** | Replicates | 10.28 | 10.77 | 9.48 | 10.78 | 9.61 | 9.49 | 10.45 | 10.18 | 10.04 |
|  |  | 10.77 | 9.44 | 9.25 | 9.29 | 10.02 | 8.68 | 10.02 | 9.59 | 9.07 |
|  |  | 9.22 | 10.32 | 9.46 | 10.82 | 10.37 | 10.09 | 9.34 | 8.08 | 9.81 |
|  | Mean | 10.09 | 10.18 | 9.397 | 10.30 | 10.00 | 9.420 | 9.94 | 9.28 | 9.64 |
|  | SEM | 0.457 | 0.391 | 0.074 | 0.504 | 0.220 | 0.409 | 0.323 | 0.625 | 0.293 |
|  | *p* _Overall_ | 0.298 | | | 0.346 | | | 0.602 | | |
|  | *p* _Control vs. Low_ | 0.983 | | | 0.860 | | | 0.576 | | |
|  | *p* _Control vs. High_ | 0.398 | | | 0.328 | | | 0.885 | | |
|  | *p* _Low vs. High_ | 0.325 | | | 0.583 | | | 0.839 | | |
| **PNW 10** | Replicates | 10.24 | 10.89 | 9.62 | 10.02 | 10.95 | 10.08 | 9.35 | 10.08 | 10.33 |
|  |  | 10.54 | 10.36 | 10.26 | 10.27 | 9.32 | 9.67 | 10.49 | 9.94 | 9.09 |
|  |  | 10.2 | 10.71 | 10.44 | 10.08 | 10.56 | 8.52 | 10.3 | 9.82 | 9.92 |
|  | Mean | 10.33 | 10.65 | 10.11 | 10.12 | 10.28 | 9.423 | 10.05 | 9.947 | 9.780 |
|  | SEM | 0.107 | 0.156 | 0.249 | 0.075 | 0.491 | 0.467 | 0.352 | 0.075 | 0.365 |
|  | *p* _Overall_ | 0.178 | | | 0.332 | | | 0.819 | | |
|  | *p* _Control vs. Low_ | 0.455 | | | 0.959 | | | 0.969 | | |
|  | *p* _Control vs. High_ | 0.681 | | | 0.467 | | | 0.806 | | |
|  | *p* _Low vs. High_ | 0.161 | | | 0.342 | | | 0.918 | | |

**Table S26**. Expression of glutamate decarboxylase in mPFC of GD 18 fetal mice measured by Western blotting.

|  | ***β -*actin** | | | **GAD65/67** | | | **Relative expression** | | |
| --- | --- | --- | --- | --- | --- | --- | --- | --- | --- |
|  | v | PFHxS - Low | PFHxS - High | Control | PFHxS - Low | PFHxS - High | Control | PFHxS - Low | PFHxS - High |
| Replicates | 12571.13 | 16566.55 | 14164.67 | 16782.91 | 10982.31 | 12421.55 | 1.335 | 0.663 | 0.877 |
|  | 16013.96 | 15758.13 | 12705.43 | 12920.67 | 15191.25 | 13731.67 | 0.807 | 0.964 | 1.081 |
|  | 17521.38 | 15435.13 | 15278.38 | 12256.96 | 12303.43 | 10360.89 | 0.700 | 0.797 | 0.678 |
| Mean | 15369 | 15920 | 14049 | 13987 | 12826 | 12171 | 0.947 | 0.808 | 0.879 |
| SEM | 1465 | 336.5 | 745 | 1411 | 1243 | 981.1 | 0.196 | 0.087 | 0.116 |
| *p* _Overall_ | 0.427 | | | 0.597 | | | 0.791 | | |
| *p* _Control vs. Low_ | 0.916 | | | 0.788 | | | 0.773 | | |
| *p* _Control vs. High_ | 0.624 | | | 0.577 | | | 0.937 | | |
| *p* _Low vs. High_ | 0.414 | | | 0.925 | | | 0.934 | | |

**Table S27**. GABA-to-glutamate ratio measured in mPFC of PNW 10 offspring mice

|  | GABA | | Glutamic acid | | GABA/Glutamic acid | |
| --- | --- | --- | --- | --- | --- | --- |
|  | Control | PFHxS-High | Control | PFHxS-High | Control | PFHxS-High |
| Replicates | 86.029 | 65.487 | 115.546 | 123.009 | 0.745 | 0.532 |
|  | 85.819 | 67.453 | 125.000 | 123.894 | 0.687 | 0.544 |
|  | 76.660 | 76.584 | 131.743 | 127.640 | 0.582 | 0.600 |
|  | 95.435 | 69.523 | 133.696 | 126.316 | 0.714 | 0.550 |
|  | 108.918 | 71.895 | 178.363 | 119.514 | 0.611 | 0.602 |
| Mean | 90.57 | 70.19 | 136.9 | 124.1 | 0.668 | 0.566 |
| SEM | 12.217 | 4.297 | 24.257 | 3.150 | 0.031 | 0.015 |
| *p* | 0.008 | | 0.276 | | 0.017 | |
