# Supplementary figures and images for: Effects of gestational perfluorohexanesulfonic acid exposure at human realistic dose on social communication deficit in mouse offspring
Source: eBioMedicine. 2026 Feb 10;125:106160. doi: 10.1016/j.ebiom.2026.106160 (PMC12914860; doi:10.1016/j.ebiom.2026.106160)

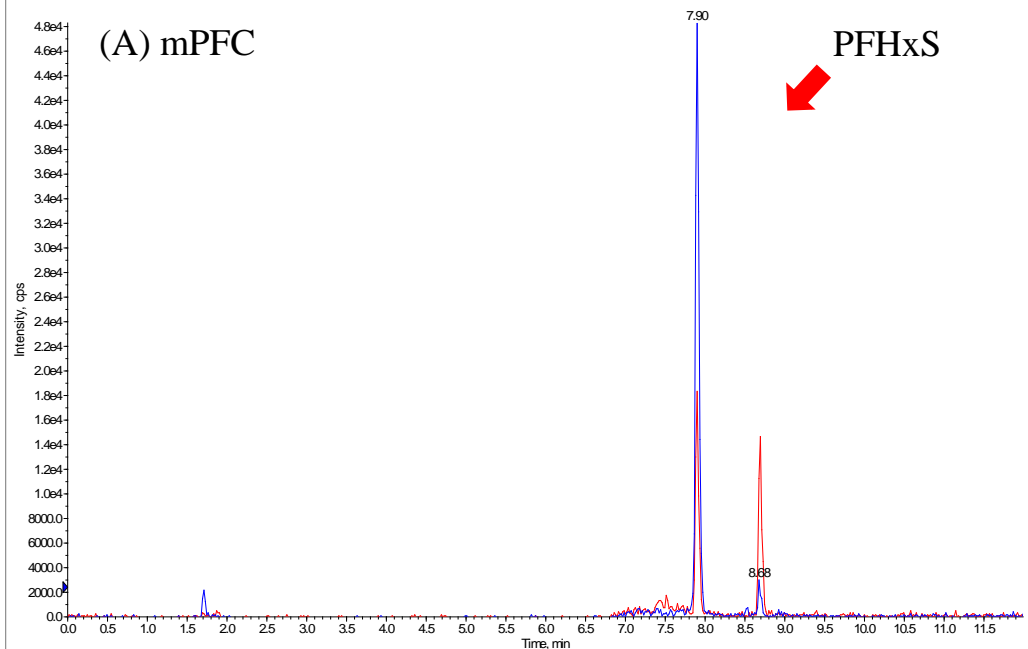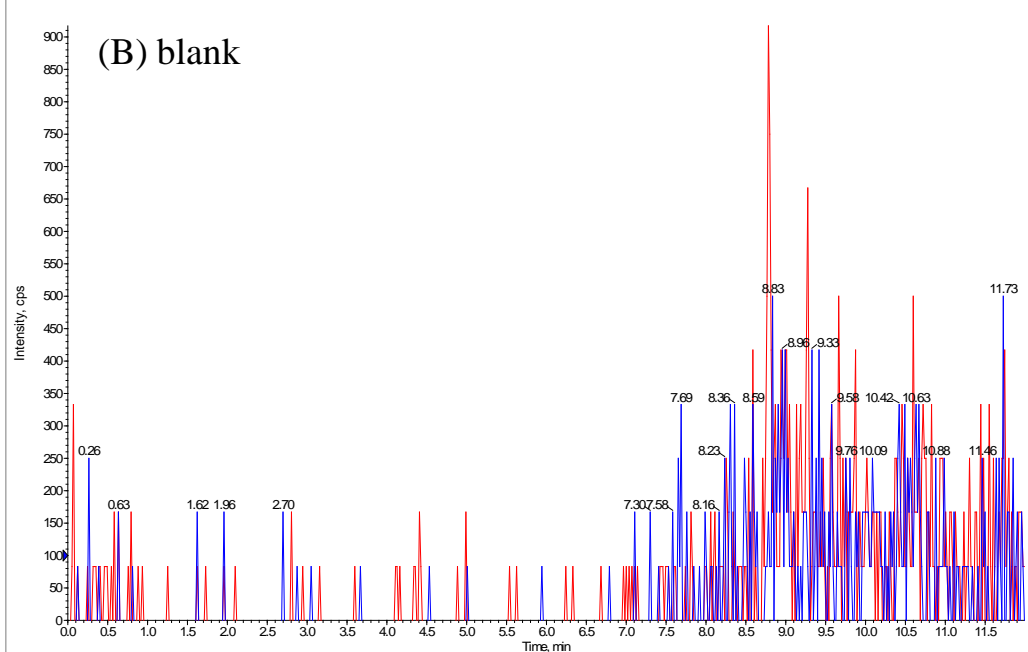

Supplement: Figure S1 [file mmc2.pdf]

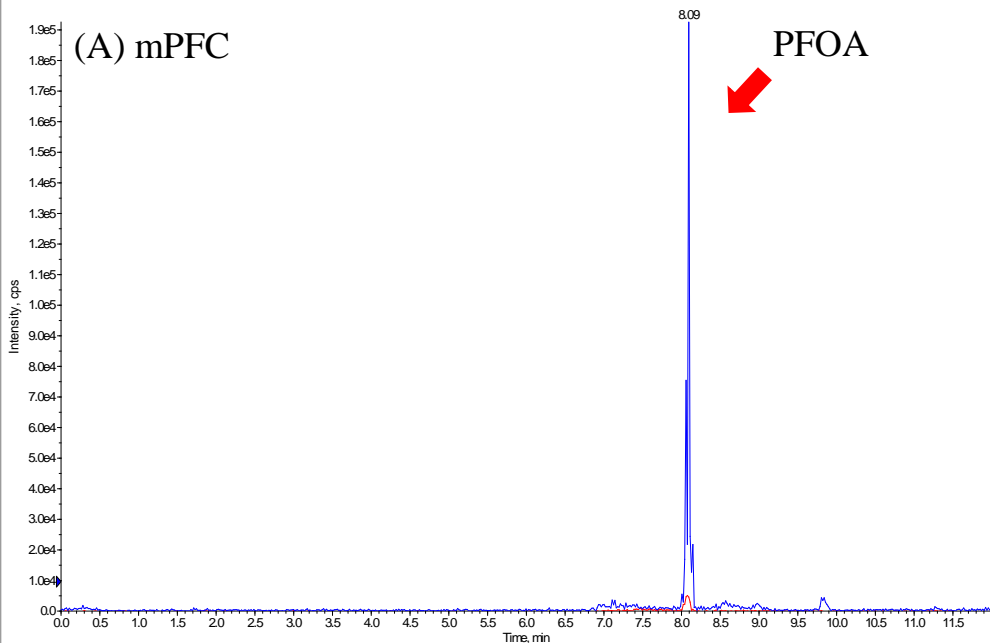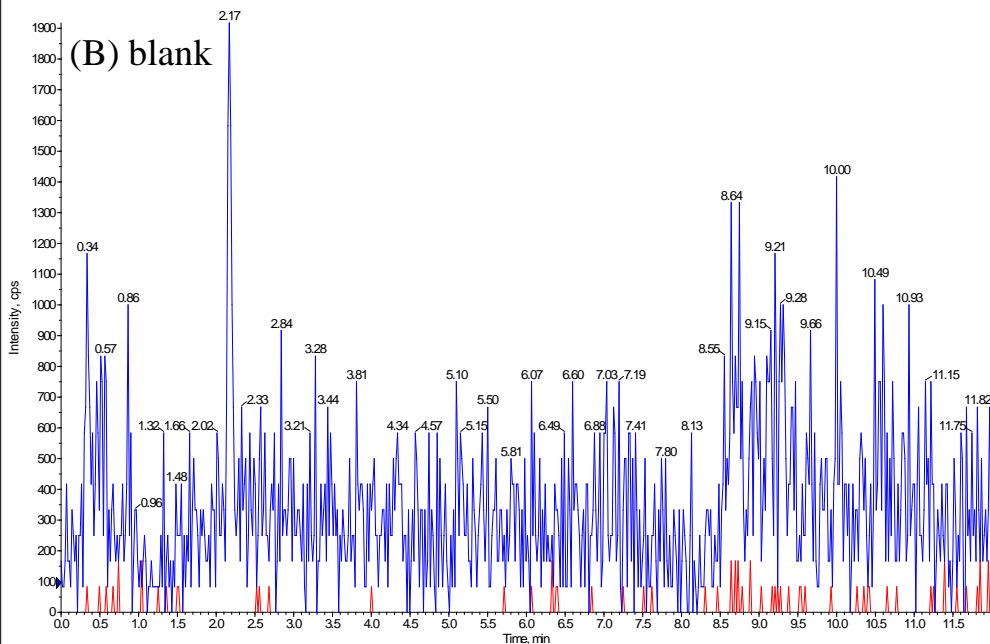

Supplement: Figure S2 [file mmc3.pdf]

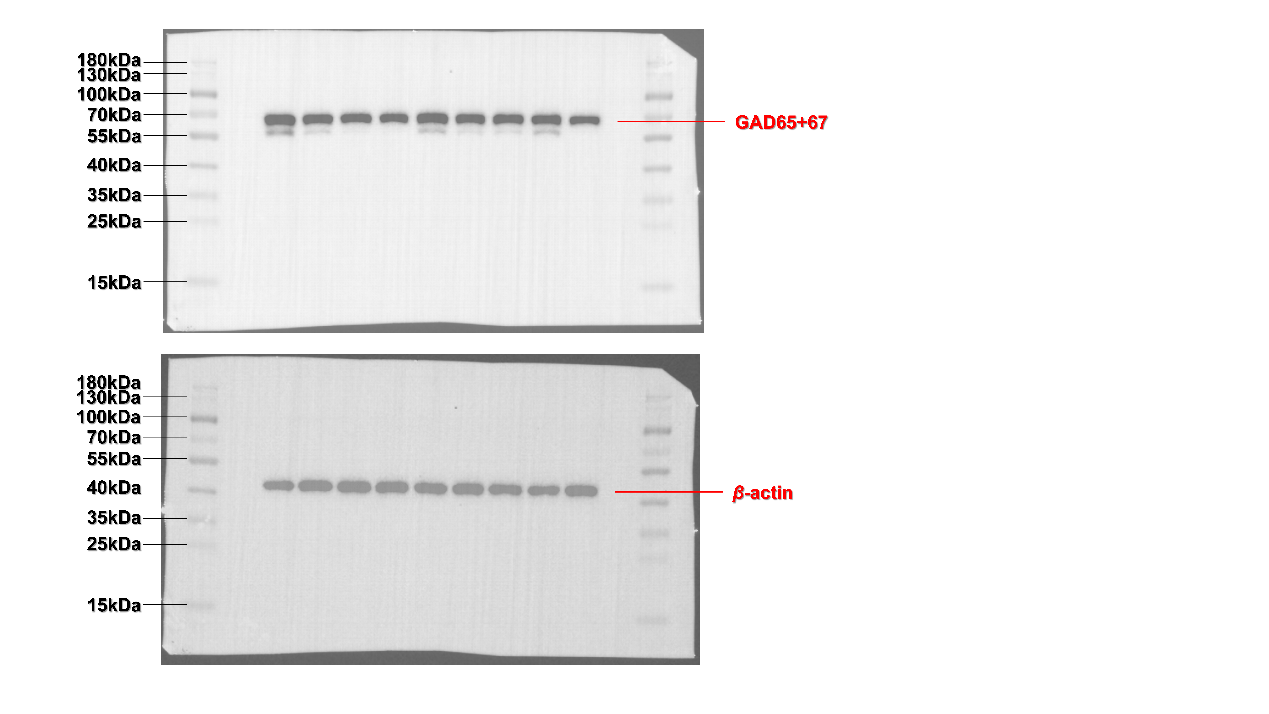
**Full Western blots**

Supplement: Supplementary Material 1 [file mmc4.docx]
