# Supplementary material for: Effects of gestational perfluorohexanesulfonic acid exposure at human realistic dose on social communication deficit in mouse offspring
Source: eBioMedicine. 2026 Feb 10;125:106160. doi: 10.1016/j.ebiom.2026.106160 (PMC12914860; doi:10.1016/j.ebiom.2026.106160)
Supplement: Supplementary Material 2 [file mmc5.pdf]

Resource Summary Report

Generated by RRID on Nov 18, 2025

Anti-GAD65 + GAD67 antibody [EPR19366]

RRID:AB_3662875
Type: Antibody

Proper Citation

Abcam Cat# ab183999, RRID:AB_3662875

Antibody Information

URL: http://antibodyregistry.org/AB_3662875

Proper Citation: Abcam Cat# ab183999, RRID:AB_3662875

Target Antigen: GAD65 + GAD67

Host Organism: Rabbit

Clonality: recombinant monoclonal

Comments: Applications: IP, WB, ICC/IF, IHC-Fr, IHC-P

Antibody Name: Anti-GAD65 + GAD67 antibody [EPR19366]

Description: This recombinant monoclonal targets GAD65 + GAD67

Target Organism: rat, mouse, human

Clone ID: EPR19366

Antibody ID: AB_3662875

Vendor: Abcam

Catalog Number: ab183999

Alternate IDs: AB_3662875

Record Creation Time: 20250820T004046+0000

Record Last Update: 20250820T085433+0000

Ratings and Alerts

No rating or validation information has been found for Anti-GAD65 + GAD67 antibody [EPR19366].

No alerts have been found for Anti-GAD65 + GAD67 antibody [EPR19366].

Data and Source Information

Source: Antibody Registry

Usage and Citation Metrics

We found 4 mentions in open access literature.

Listed below are recent publications. The full list is available at RRID.

Wang Y, et al. (2025) Septo-subicular cholinergic circuit promotes seizure development via astrocytic inflammation. Cell reports, 44(5), 115712.

Jansen MI, et al. (2025) Neuronal PAC1 deletion impairs structural plasticity. Life sciences, 378, 123843.

Gao Y, et al. (2025) VIVIT: Resolving trans-scale volumetric biological architectures via ionic glassy tissue. Cell.

Lefner MJ, et al. (2025) Flexible updating of reward and punishment contingencies by VTA GABA neurons. Current biology : CB, 35(16), 3973.
